# Pollution gradients shape microbial communities associated with *Ae. albopictus* larval habitats in urban community gardens

**DOI:** 10.1093/femsec/fiae129

**Published:** 2024-09-26

**Authors:** Penelope Duval, Edwige Martin, Laurent Vallon, Pierre Antonelli, Maxime Girard, Aymeric Signoret, Patricia Luis, Danis Abrouk, Laure Wiest, Aurélie Fildier, Christelle Bonnefoy, Patrick Jame, Erik Bonjour, Amelie Cantarel, Jonathan Gervaix, Emmanuelle Vulliet, Rémy Cazabet, Guillaume Minard, Claire Valiente Moro

**Affiliations:** Universite Claude Bernard Lyon 1, Laboratoire d'Ecologie Microbienne, UMR CNRS 5557, UMR INRAE 1418, VetAgro Sup, 69622 Villeurbanne, France; Universite Claude Bernard Lyon 1, Laboratoire d'Ecologie Microbienne, UMR CNRS 5557, UMR INRAE 1418, VetAgro Sup, 69622 Villeurbanne, France; Universite Claude Bernard Lyon 1, Laboratoire d'Ecologie Microbienne, UMR CNRS 5557, UMR INRAE 1418, VetAgro Sup, 69622 Villeurbanne, France; Universite Claude Bernard Lyon 1, Laboratoire d'Ecologie Microbienne, UMR CNRS 5557, UMR INRAE 1418, VetAgro Sup, 69622 Villeurbanne, France; Universite Claude Bernard Lyon 1, Laboratoire d'Ecologie Microbienne, UMR CNRS 5557, UMR INRAE 1418, VetAgro Sup, 69622 Villeurbanne, France; Universite Claude Bernard Lyon 1, Laboratoire d'Ecologie Microbienne, UMR CNRS 5557, UMR INRAE 1418, VetAgro Sup, 69622 Villeurbanne, France; Universite Claude Bernard Lyon 1, Laboratoire d'Ecologie Microbienne, UMR CNRS 5557, UMR INRAE 1418, VetAgro Sup, 69622 Villeurbanne, France; Universite Claude Bernard Lyon 1, Laboratoire d'Ecologie Microbienne, UMR CNRS 5557, UMR INRAE 1418, VetAgro Sup, 69622 Villeurbanne, France; Univ Lyon, CNRS, Université Claude Bernard Lyon 1, Institut des Sciences Analytiques, UMR 5280, 5 Rue de la Doua, F-69100 Villeurbanne, France; Univ Lyon, CNRS, Université Claude Bernard Lyon 1, Institut des Sciences Analytiques, UMR 5280, 5 Rue de la Doua, F-69100 Villeurbanne, France; Univ Lyon, CNRS, Université Claude Bernard Lyon 1, Institut des Sciences Analytiques, UMR 5280, 5 Rue de la Doua, F-69100 Villeurbanne, France; Univ Lyon, CNRS, Université Claude Bernard Lyon 1, Institut des Sciences Analytiques, UMR 5280, 5 Rue de la Doua, F-69100 Villeurbanne, France; Univ Lyon, CNRS, Université Claude Bernard Lyon 1, Institut des Sciences Analytiques, UMR 5280, 5 Rue de la Doua, F-69100 Villeurbanne, France; Universite Claude Bernard Lyon 1, Laboratoire d'Ecologie Microbienne, UMR CNRS 5557, UMR INRAE 1418, VetAgro Sup, 69622 Villeurbanne, France; Universite Claude Bernard Lyon 1, Laboratoire d'Ecologie Microbienne, UMR CNRS 5557, UMR INRAE 1418, VetAgro Sup, 69622 Villeurbanne, France; Univ Lyon, CNRS, Université Claude Bernard Lyon 1, Institut des Sciences Analytiques, UMR 5280, 5 Rue de la Doua, F-69100 Villeurbanne, France; UMR 5205, Laboratoire d'Informatique en image et systèmes d'information, Université de Lyon, Villeurbanne, France; Universite Claude Bernard Lyon 1, Laboratoire d'Ecologie Microbienne, UMR CNRS 5557, UMR INRAE 1418, VetAgro Sup, 69622 Villeurbanne, France; Universite Claude Bernard Lyon 1, Laboratoire d'Ecologie Microbienne, UMR CNRS 5557, UMR INRAE 1418, VetAgro Sup, 69622 Villeurbanne, France

**Keywords:** *Aedes albopictus*, microbial communities, organic molecules, physicochemical parameters, pollution, urbanization, water

## Abstract

The Asian tiger mosquito *Aedes albopictus* is well adapted to urban environments and takes advantage of the artificial containers that proliferate in anthropized landscapes. Little is known about the physicochemical, pollutant, and microbiota compositions of *Ae. albopictus*-colonized aquatic habitats and whether these properties differ with noncolonized habitats. We specifically addressed this question in French community gardens by investigating whether pollution gradients (characterized either by water physicochemical properties combined with pollution variables or by the presence of organic molecules in water) influence water microbial composition and then the presence/absence of *Ae. albopictus* mosquitoes. Interestingly, we showed that the physicochemical and microbial compositions of noncolonized and colonized waters did not significantly differ, with the exception of N_2_O and CH_4_ concentrations, which were higher in noncolonized water samples. Moreover, the microbial composition of larval habitats covaried differentially along the pollution gradients according to colonization status. This study opens new avenues on the impact of pollution on mosquito habitats in urban areas and raises questions on the influence of biotic and abiotic interactions on adult life-history traits and their ability to transmit pathogens to humans.

## Teaser

Microbial composition of larval habitats covaried differentially along the pollution gradients according to the presence/absence of larvae. Noncolonized water samples exhibited higher concentrations of N_2_O and CH_4_.

Abbreviations dbRDA: Distance-based redundancy analyses
C: Colonized water samples
L: Larvae
Lmm: Linear mixed model
NC: Noncolonized water samples
OTU: Operational taxonomic unit
PLS-DA: Partial least squares discriminant analysis

## Introduction

Roughly 3% of the Earth’s land surface is occupied by urban areas (CIESIN et al. 2011). The rapid rise in the size, density, and heterogeneity of urban areas has had deep impacts on urban populations, biodiversity, and climate (Vlahov 2002, Li et al. 2022, Khanh et al. 2023). Rapid urban expansion has led to an increase in poverty, social inequality, temperature [through the urban heat island—(UHI) effect], and homogenization of biodiversity and has also generated much waste and pollution (Kalnay and Cai 2003, McKinney 2006, Dociu and Dunarintu 2012, Liddle 2017, Liang et al. 2019). A promising solution for reducing the negative impacts of urbanization is the creation of green areas and addition of vegetation in cities (Susca et al. 2011, Gago et al. 2013, Pascal et al. 2019). Urban green areas are efficient in remedying UHIs by cooling effects in cities (Norton et al. 2015). In addition, vegetation and green spaces have been shown to reduce atmospheric pollution, and have had a positive impact on human health by attenuating mortality and improving mental health through increasing physical activities (Lepczyk et al. 2017, Kondo et al. 2018, Nunho Dos Reis et al. 2022, Royer et al. 2023). Green urban spaces include different types ranging from parks and gardens to cemeteries and derelict lands (Catalano et al. 2021). Among them, community gardens are increasingly implemented in European cities (Ochoa et al. 2019). The concept of the family garden (formerly known as workers’ gardens) emerged during the Industrial Revolution, in a period of intense urbanization to mitigate the problem of the precariousness of workers (Keshavarz et al. 2016). Currently, diverse types of community gardens exist, e.g. family gardens, shared gardens, and integrated gardens. These urban gardens are managed by different organizations, including metropolises, municipalities, private organizations, and nonprofit associations (Holland 2004).

Urban community gardens play a crucial role in promoting biodiversity (Jha et al. 2023). They often feature a variety of plant species that attract pollinators such as bees, butterflies, and other beneficial insects, ensuring a continuous supply of nectar and pollen for both bee and nonbee species (Schmack and Egerer 2023). This supports the health of urban ecosystems and facilitates the pollination of nearby plants and crops. For example, a study in Dunedin, southern New Zealand, found Collembola, Amphipoda, and Diptera to be the most abundant taxa in 55 domestic gardens (Baratt et al. 2015). Mosquitoes, like other insects, are attracted to and feed on both floral and extrafloral nectar as an energy source (Jhumur et al. 2008, Nyasembe and Torto 2014). While male mosquitoes need nectar to survive, the sugars from nectar help female mosquitoes increase their life expectancy, survival rate, and reproduction (Foster 1995). Interestingly, recent observations suggest that mosquitoes could also act as pollinators (Lahondere et al. 2020). In addition to the presence of diverse plant species, urban community gardens often contain a high density and diversity of still water containers (e.g. watering cans, flower buddies, or rainwater collectors), which are suitable oviposition habitats for mosquitoes, including the Asian tiger mosquito (Hayden et al. 2010, Duval et al. 2022). Oviposition site selection is a critical behavior that influences egg hatching, juvenile development rate, and larvae or pupae survival. To locate floral scents and suitable oviposition sites, mosquitoes utilize a highly sensitive olfactory system that integrates visual, gustatory, and olfactory cues (Bentley and Day 1989, Barredo and DeGennaro 2020). Females can identify aquatic habitats conducive to the development and survival of their offspring based on characteristics such as color, size, and sunlight, assessing water quality through olfactory and tactile cues (Day 2016). Recent studies have also highlighted the role of microbe-derived volatiles as environmental cues influencing mosquito foraging and oviposition behavior decisions (Afify and Galizia 2015, Girard et al. 2021, Peach et al. 2021, Sobhy and Berry 2024).

The proliferation of the Asian tiger mosquito *Aedes albopictus* constitutes a major public health challenge due to its ability to transmit more than 19 arboviruses, such as dengue, Zika, and chikungunya, in humans (Paupy et al. 2009, Duval et al. 2023). Previous studies demonstrated that the number of larval habitats and the development and survival rates of *Ae. albopictus* were positively impacted by the urban context (Li et al. 2014, Wilke et al. 2019, Westby et al. 2021). In urban areas, this species breeds in human-made containers, where water could be exposed to various sources of pollutants. The biotic and abiotic characteristics of larval habitats determine the choice by gravid females for oviposition and obviously affect the development of offspring, from larvae to adults (Afify and Galizia 2015, Malassigné et al. 2020, Hery et al. 2021, Dalpadado et al. 2022). For instance, physicochemical parameters such as pH, temperature, organic matter, phosphate, ammonia, and potassium are known to guide females to choose egg-laying sites (Darriet 2019). In addition, microbial communities produce volatile organic compounds that can be attractive or repulsive to mosquitoes (Weisskopf et al. 2021). More globally, the microbial structure in breeding sites partly shapes the larval microbiota and can have carryover effects on the physiology of adults and their ability to transmit pathogens (Dickson et al. 2017, Guégan et al. 2018, Zheng et al. 2023). However, reciprocal interactions between physicochemical parameters and microbial structure in artificial breeding sites and their impact on the mosquito life cycle are still poorly understood.

In mainland France (Europe), since the first identification of autochthonous cases of dengue in 2010, viral diseases transmitted by mosquitoes have tended to increase each year, as well as the number of regions at risk due to the continuous spread of the Asian tiger mosquito (Cochet et al. 2022). Following previous observations, the aim of this study was to evaluate whether and how human activities, particularly pollution gradients, impact the biotic and abiotic properties of *Ae. albopictus* larval breeding sites in community gardens in the Lyon metropolis, one of the largest and most populous cities in mainland France. This study mainly focused on containers mainly filled with rainwater but also occasionally supplemented with tap water, both of which may sometimes contain pollutants. For this purpose, we analysed the physicochemical and microbiological composition of water by comparing *Ae. albopictus*-colonized and *Ae. albopictus*-noncolonized waters as well as the microbiota composition of larvae. Microbial communities were analysed by characterizing bacterial and microeukaryotic communities through high-throughput sequencing. Physicochemical water properties (i.e. pH, temperature, conductivity, oxidation‒reduction potential, turbidity, and carbon content) and organic molecules were also characterized by using multiparameter probes, gas and ion chromatography, and liquid chromatography coupled with high-resolution mass spectrometry (LC–HRMS). We then evaluated whether pollution gradients (characterized either by water physicochemical properties combined with pollution variables reflecting proximity to polluted areas or by organic molecule presence in water) influence water microbial composition depending on the presence/absence of *Ae. albopictus* mosquitoes. While microbial and physicochemical compositions were very similar between colonized and noncolonized water samples, our results revealed differential effects of pollution gradients on microbial community structure according to water colonization status.

## Experimental procedures

### Selection and delineation of the study areas

By taking advantage of our recent database, in which we have inventoried a total of 288 community gardens in the metropolitan area of Lyon (Duval et al. 2022), we characterized the gardens based on different criteria: the proximity to different pollution sources, the surface of nearby agricultural areas, and the level of atmospheric pollution. The proximity with highways and agricultural lands were defined thanks to the BD CARTO^®^ of French national mapping agency (www.geoportail.gouv.fr), while that of industrial areas was characterized using an open platform for French public data (www.data.gouv.fr). Highways are significant sources of air pollution, including particulate matter, nitrogen oxides, and other pollutants. Therefore, normalization for proximity to a highway was considered, as it accounts for the significant pollution impacts highways may have on urban gardens.

The surface of agricultural areas was calculated from satellite images of Google Earth using QGIS (www.qgis.org). In addition, air pollution through emission data of NO_2_ and particular matter (PM_2.5_ and PM_10_) was provided by Atmo Auvergne-Rhône-Alpes (www.atmo-auvergnerhonealpes.fr) the local authority for air quality as 1 × 1 grid, in 2021.

While ensuring geographical representation across Lyon metropolis would have been ideal for capturing the full range of conditions affecting urban community gardens, sites were selected if they met the following conditions: proximity to one pollution source (atmospheric, industrial, or agricultural) (Table 1), surface area >1 km², availability of access authorization, and presence of *Ae. albopictus*-colonized and noncolonized waters.

**Table 1.** Characterization of selected community gardens. For each garden, the level of atmospheric pollution, distance from the garden to each pollution source (agricultural, industrial, and atmospheric pollution), and calculated pollution variables (Var_indus, Var_agri, and Var_atmo) are given.

	Pollution group	Atmospheric pollution scores	Distance from the garden to each pollution source (m)			Calculated pollution variables		
Garden			Agricultural	Industrial	Atmospheric	Var_indus	Var_agri	Var_atmo
MID	AGRI	8	2624	1167	183	0.0009	4993	0.0437
MOU	INDUS	6	1311	1829	544	0.0005	386	0.011
PER	AGRI	5	158	3442	121	0.0003	228	0.0209
RECU	ATMO	12	1228	4598	4	0.0002	1365	0.1958
REB	ATMO	12	2624	1986	56	0.0005	191	0.2159
BIL	ATMO	12	4066	1818	26	0.0004	92	0.1534
GAR	AGRI	6	327	2209	476	0.0005	1144	0.011
ARK	INDUS	8	2640	528	500	0.0019	192	0.0152
ALST	ATMO	8	1549	3967	464	0.0003	80	0.0172
FRA	INDUS	3	1584	787	3068	0.0013	48	0.0009
TAS	INDUS	6	2044	802	1587	0.0012	923	0.0038
JUST	ATMO	12	3541	2999	24	0.0003	5	0.0587
DEC	AGRI	4	200	3422	59	0.0003	246	0.0382
FOR	ATMO	12	2759	2022	23	0.0005	182	0.2352
SYT	INDUS	11	3317	1974	204	0.0005	113	0.0538
QUA	INDUS	3	1545	590	2495	0.0017	585	0.0012
COR	AGRI	5	164	8643	1430	0.0001	193	0.0034
ESP	AGRI	9	669	1963	814	0.0005	559	0.0111
EDF	INDUS	12	7059	631	14	0.0016	2508	0.0147
BIG	AGRI	3	465	1457	620	0.0007	1099	0.0044
AVIA	AGRI	6	319	1491	298	0.0007	1659	0.0148
BONN	AGRI	6	682	1373	220	0.0007	771	0.0209
VOIL	ATMO	12	3371	2288	16	0.0004	560	0.3004

Pollution variables reflecting each pollution source i.e. atmospheric (Var_atmo), agricultural (Var_agri), or industrial pollution (Var_indus) were then calculated for each garden using the following formula:


\begin{eqnarray*}
Var\_atmo = \ \frac{{qN{{o}_{2\ }} + \ qP{{M}_{2.5}} + qP{{M}_{10}}\ }}{{dH}},
\end{eqnarray*}

where “qNO_2_,” “qPM_2.5_,” and “qPM_10_” correspond to air pollution scores ranging from 1 (the first quartile of gardens with the lowest pollution rates) to 4 (the last quartile of gardens with the highest pollution rates) and “dH” corresponds to the distance between the garden and the nearest highway.


\begin{eqnarray*}
Var\_agri\ = \frac{{\textit{surfac}{{e}^2}}}{{dA}},
\end{eqnarray*}

where “surface” corresponds to the area of the nearest agricultural area and “dA” corresponds to the distance between the garden and the nearest agricultural area.


\begin{eqnarray*}
Var\_indus\ = \ \frac{1}{{dI}},
\end{eqnarray*}

where “dI” corresponds to the distance between the garden and the nearest industrial area.

Atmospheric, industrial, and agricultural pollution variables were normalized for each community garden using the following formula:


\begin{eqnarray*}
\frac{{X - min}}{{max - min}},
\end{eqnarray*}

where “X” corresponds to calculated pollution variables and “min” and “max” correspond to the minimum and maximum variables for the same pollution context.

### Sample collection

**Figure 1.**
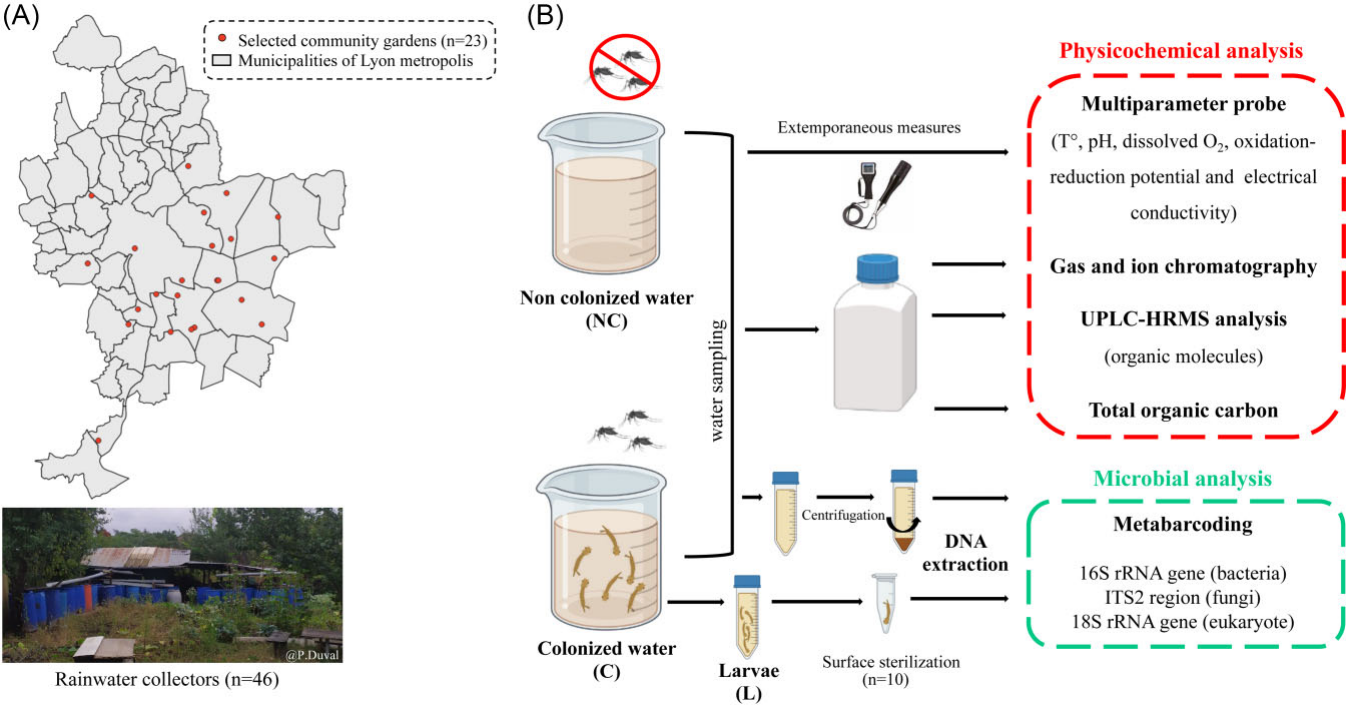
Sample collection overview. (A) Distribution of community gardens in the Lyon metropolis, outlining the sites where water sampling collection was performed. The map of the Lyon metropolis was created using QGIS (www.qgis.org). (B) Sample processing describing physicochemical and microbial analyses of selected samples.

Field sampling was performed in 23 community gardens of Lyon metropolis between June and September 2021. To minimize the influence of precipitation on pollution dispersion and mosquito dynamics, sampling was conducted only after ensuring no rainfall had occurred in the previous 48 h. Samples were collected in the morning within the same time frame, with air temperatures consistently similar. For each garden, *Ae. albopictus* larval habitats were sampled by collecting water from a colonized and a noncolonized reservoir (referred hereafter as C and NC, respectively) as well as *Ae. albopictus* third and fourth instar larvae (*n* = 50 per breeding site) (referred hereafter as L). Colonized waters were identified by the presence of both *Ae. albopictus* larvae in water and adults flying around the reservoir. Larvae were identified using morphological identification keys of Darsie and Ward (1981) (Kline et al. 2006) and then verified by COI barcoding by randomly selected four larvae per colonized water, as previously described (Raharimalala et al. 2012). Polymerase chain reaction (PCR) products were sent to Sanger sequencing at Microsynth France SAS (Vaulx-en-Velin). Regarding noncolonized waters, the entire volume of water in the container was filtered to ensure the absence of larvae. For each selected container (C and NC), water was mixed thoroughly before sampling and the water temperature (°C), pH, dissolved oxygen (mg/l), oxidation–reduction potential (mV), and electrical conductivity (μS/cm) were measured directly in the field with a portable multiparameter water probe (Horiba, U-50, France). Water samples (1 l) were collected and split into one sterile 50 ml Falcon tube (Greiner, Germany) for microbial analysis (40 ml) and three HDPE bottles (VWR, Radnor, USA) for chemical analysis (400 ml, 250 ml, and 250 ml), respectively. Third- and fourth-instar larvae were collected with a sterile plastic pipette into a sterile 50 ml Falcon tube (Greiner) and then transferred individually to the laboratory in sterile 1.5 ml microcentrifuge tubes (Sarstedt, Germany). Water samples were stored at −20°C and defrosted before each analysis except for ion chromatography analyses where samples were filtered at 0.22 µm before freezing. The process of field water sampling is detailed in Fig. 1.

### DNA extraction

Water samples were homogenized by vortexing for 1 min and a 2 ml aliquot was centrifuged for 10 min at maximum speed (13 400 r/m) at 4°C. The supernatant was discarded and a new aliquot of 2 ml was added. This step was repeated three times for each sample. Pooled pellets were resuspended in 250 µl of CTAB buffer (2% hexadecyltrimethyl ammonium bromide, 1.4 M NaCl, 0.02 M EDTA, 0.1 M Tris pH8, and 0.2% 2-β mercaptoethanol). After incubation for 1 h at 60°C in a shaker (300 r/m), 4 µl of RNase (100 mg/ml) was added and incubated at 37°C for 5 min. Mixtures of phenol:chloroform:isoamyl alcohol (25:24:1) and chloroform:isoamyl alcohol (24:1) were added and after vortexing for 5 min, samples was centrifuged for 30 min at 13 200 r/m at room temperature, DNA was precipitated using isopropyl alcohol and Glycoblue coprecipitant (15 mg/ml). DNA pellets were rinsed with 75% cold ethanol, air-dried, and resuspended in 24 µl of sterile RNase-free water. Prior to DNA extraction, larvae were surface-sterilized as previously described (Zouache et al. 2022). Each larva was crushed for 1 min using a FastPrep-24™ 5 G (MP Biomedical) into a sterile 2 ml screw-cap tube containing three beads of 3 mm diameter (Merck) and 250 µl of CTAB buffer. Larvae DNA extractions were then performed as previously described for water samples. DNA extractions from larvae and water were performed in 23 batches (one DNA extraction per garden) and a negative control (i.e. extraction performed without biological matrix) was included in each batch. DNA extracts purity and concentration were estimated using the NanoPhotometer NP80 (Implen) and the Qubit dsDNA High Sensibility Kit combined with the Qubit 4 fluorometer (Invitrogen), respectively.

### Library preparation and high throughput sequencing

DNA samples from water and larvae were used as templates to systematically amplify three gene markers. The V5–V6 region of the 16S rRNA genes of bacteria was amplified using the universal primers 784F/1061R (Andersson et al. 2008), whereas the V1–V2 region of the 18S rRNA genes of eukaryotes was amplified using the universal primers Euk82F/Euk516R (Thongsripong et al. 2018) (Table S1). The fungal nuclear ribosomal internal transcribed spacer (ITS2 region) was amplified using the universal primers gITS7/ITS4 (Ihrmark et al. 2012) (Table S1). PCR amplifications were also performed on 10 larvae per garden for bacterial and fungal markers. Primers were tagged with the Illumina adapters 5′-GTC TCG TGG GCT CGG AGA TGT GTA TAA GAG ACAG-3′ and 5′-TCG TCG GCA GCG TCA GAT GTG TAT AAG AGA CAG-3′, enabling a two-step PCR construction of amplicon libraries. PCR amplifications were conducted in duplicate in a Bio-Rad T1000 thermal cycler (Bio-Rad, Hercules, USA) with 5 × HOT BioAmp Master Mix (Biofidal, France) containing 2 µl sample DNA and 1 µM of each primer as previously described (Zouache et al. 2022, Girard et al. 2023) (Table S2). For recalcitrant samples, PCR additives such as 10 × of GC rich enhancer and 0.5 mg/ml of bovine serum albumin (New England Biolabs, Evry, France) were added to the PCR mix. Amplicons were checked by electrophoresis on 1.5% agarose 20 min at 100 V and UV visualization. The PCR products were then sent to Microsynth sequencing company (Balgach, Switzerland) for purification and second-step PCR, 2 × 300 bp Miseq sequencing (Illumina). DNA matrix that did not amplify during the first PCR reaction was used as templates for quantitative PCR, as previously described (Girard et al. 2023).

### Bioinformatics analysis

A total of 7 325 894, 9 591 794, and 2 311 285 reads were obtained for each dataset (i.e. bacterial 16S rRNA genes, fungal ITS2, and eukaryotic 18S rRNA genes, respectively) and paired-end reads were demultiplexed in the different samples. R1 and R2 reads were merged using Fastp, based on five mismatches in the overlap region. Sequence quality control and analysis of sequence data were carried out using the FROGS pipeline (Escudié et al. 2018). Briefly, denoising was carried out by discarding reads without the expected length of that displayed ambiguous bases (N). Clustering was performed using SWARM (Mahé et al. 2014) based on an aggregation distance of 3 for identification of operational taxonomic units (OTUs). Chimeric OTUs were discarded using VSEARCH (Rognes et al. 2016) and sequences of low abundance were filtered at 0.005% of all sequences (Bokulich et al. 2013). The alignment and OTU affiliation were performed using Silva v.138.1 database for bacteria and eukarya (Quast et al. 2012) and the UNITE 8.2 database for fungi (Nilsson et al. 2019). Reads that did not align were filtered out and contaminant OTUs were filtered out using negative control (negative control of extraction and PCR). OTUs were removed if they were detected in the negative QC and their relative abundance was not at least 10 times greater than that observed in the negative control (Minard et al. 2015). Normalization for sample comparison was performed by randomly resampling down to 4103, 6567, and 7204 sequences in the bacterial, fungal, and eukaryotic datasets, respectively.

### Gas and ion chromatography

For gas chromatography analysis, water samples were thawed at room temperature then transferred into internal flask gases and 50 ml of helium was added. The flasks were incubated at 60°C for 3 h at 150 r/m. After incubation, gas present in water samples were measured using a gas chromatograph (µGC R990, SRA Instrument, Marcy L’Etoile, France) with MS5A SS columns TCD detector (10 m × 0.25 mm × 30 µm BF) and PPQ (µm 10m × 0.25 mm × 8 µm) and 48 channels custom automatic sample changer (SRA Instrument). A calibration mix solution was prepared using helium combined with each dissolved gas, with concentrations ranging from 0 to 14 000 ppm for CO_2_, 0 to 50 000 ppm for N_2_O, 0 to 100 000 ppm for CH_4_, 0% to 79% for N_2_, and 0% to 21% for O_2_. The ions present in water samples were analysed by an ionic chromatograph (AQUION) with automatic sample changer (AS-AP 120). Anion concentrations were measured on precolumn and column (AS9-HC, 4 × 250 mm) at 30°C and eluted with 9 mM Na_2_CO_3_ with a flow rate of 1 ml/min and a 4-mm carbonate suppressor (AERS 500). Cation concentrations were evaluated on precolumn and column (CS-19, 4 × 250 mm) at 40°C and eluted with 30 mM methanesulfonic acid with a flow rate of 1 ml/min and a 4-mm suppressor (CDRS 600). Both ions were detected with a conductimeter (Thermofischer Electron SAS Courtaboeuf 91941). Calibration standards were prepared for each ion with concentrations ranging from 0 (ultrapure water only) to 500 mg/l. Anions (F^−^, Cl^−^, NO_2_^−^, Br^−^, NO_3_^−^, PO_4_^3−^, and SO_4_^3−^) were prepared by diluting a more concentrated stock solution (1 g/l) from NaF, KCl, NaNO_2_, KBr, KNO_3_, Na_3_PO_4_.12H_2_O, and K_2_SO_4_. Cations (Na^+^, NH_4_^+^, K^+^, Mg^2+^, and Ca^2+^) were prepared by diluting a more concentrated stock solution (1 g/l) from NaCl, (NH_4_)_2_SO_4_, KCl, MgCl_2_.6H_2_O, and CaCl_2_.2H_2_O.

### UPLC–HRMS analysis

In these analyses, the 23 gardens were classified into three groups according to their proximity to different pollution sources and the presence of atmospheric pollutants (Table 1). An aliquot of each water sample (5 ml) was prepared by liquid–liquid extraction with 5 ml acetonitrile acidified with 0.1% formic acid. Each sample was fortified with a labeled internal standard (diuron-d6), then ultrasonicated, centrifuged, and frozen overnight at −18°C. A 2.5-ml aliquot was retrieved. A volume of 500 µl of each sample extract was pooled to prepare a quality control sample (QC). Then the 2 ml left extracts were dried under a slight nitrogen flow at 40°C. Each dry extract was reconstituted in 200 µl of water/methanol 90/10 (v/v). Moreover, to ensure the reliability of the results, each sample was extracted and analysed three times. Extracts with ultrapure water were prepared with the same protocol and considered as blank matrix. Analyses by LC–HRMS were performed on an Ultimate 3000 UHPLC system (Thermo Scientific®, MA, USA) coupled to a quadrupole time-of-flight mass spectrometer (QToF) (Maxis Plus, Bruker Daltonics®, Bremen, Germany) equipped with an electrospray ionization interface. Analyses were carried out in reverse phase (elution gradient) employing an Acclaim RSLC 120 C18 column (2.2 μm, 100 × 2.1 mm; ThermoScientific®), protected with a KrudKatcher Ultra In-Line Filter guard column from Phenomenex (Torrance, CA, USA) and heated at 30°C. The injected volume was 5 μl. Mobile phases consisted of: an aqueous phase (90%/10% ultrapure water/methanol mixture with 5 mM ammonium formate and 0.01% formic acid) and an organic phase (methanol with 5 mM ammonium formate and 0.01% formic acid). All extracts were analysed in positive electrospray ionization with the following settings: capillary voltage of 3600 V, end plate offset of 500 V, nebulizer pressure of 3 bar (N_2_), drying gas of 9 l/min (N_2_), and drying temperature of 200°C. An external calibration of exact masses was systematically performed at the beginning of each run, using a solution of sodium formate and acetate (0.5 ml of 1 M NaOH, 25 μl of formic acid, and 75 μl of acetic acid in in H_2_O/isopropanol 50/50, v/v), generating cluster ions [M+H] + in the range 90.9766–948.8727 Da with high precision calibration (HPC) mode at a search range ± 0.05 m/z. Accepted standard deviations were inferior to 0.5 ppm. QCs were run several times at the beginning of the analytical sequence to equilibrate the column and at regular intervals throughout the sequence to check for instrument drift and to control analytical repeatability and sensitivity. Instrument control, data acquisition, and processing were performed using OTOF Control and HystarTM (v4.1, Bruker Daltonics®) software. Samples were randomly analysed in full-MS scan over a m/z range of 50-1000 Da. QCs were also analysed in data-dependent analysis, a mode with alternative acquisition in MS and MSMS, with predefined parameters. The data preprocessing was performed with W4M and Metaboscape (Giacomoni et al. 2015). A feature matrix was generated for each sequence. It contained 1382 features for which the mass to charge ratio m/z in ion adduct form, the retention time (RT) of each detected peak, the intensity in each sample. Each feature was then normalized to the intensity of diuron-d6. It was discarded from the dataset when the coefficient of variation in the QCs was over 30%. Missing intensities in a replicate were replaced by the mean intensities in the two others replicate or by 0 if not available. Then for each group of gardens (Table 1), a detection rate was calculated. If it was less than 100%, the feature was not considered afterwards. Signals that were not significantly higher in samples compared to blanks (intensity ratio >10), and were not significantly different between C and NC (*t*-test; *P* > .05) were not considered. Annotation and identification were performed using Metaboscape and MetFrag (Wolf et al. 2010) an *in silico* fragmentation tool, by comparing experimental MSMS spectra. Using these filters, a list of 147 LC–HRMS signals was kept on the 1382 features. Finally markers were putatively identified according to the level of identification confidence from 1 to 5 (Schymanski et al. 2015) (Table 2).

**Table 2.** Putative identification of organic molecules in noncolonized and colonized water samples.

	Exact mass m/z (Da)			Level of identification confidence		Detection in water samples	
Id micropollutant		tR (min)	Formula		Micropollutant affiliation	C	NC
M181T564	181.1225	9.4	C11H16O2	2B	3-tert-butyl-4-hydroxyanisole	VOIL, JUST, ARK, FRA, BIL	SYT, FRA, EDF, ALST, TAS
M197T437	197.1175	7.28	C11H16O3	2A	Loliolide	VOIL, ARK, JUST, FRA, BIL	SYT, QUA, FRA, ALST, VOIL
M227T64	227.0867	1.07	C5H12N6O3	2B	Dimethylenetriurea	ARK, GAR	PER
M233T692	233.154	11.53	C15H20O2	2A	Costunolide, indicanone, alantolactone, eremofrullanolide, glechomanolide, frullanolide	ARK	
M277T743	277.2165	12.38	NA	5	Familly of phtalates	VOIL	FOR, QUA, TAS, GAR
M279T761	279.2321	12.68	C18H32O3	2B	13(S)-HODE, alpha-dimorphecolic acid laetisaric acid 9(10)-EpOME, (+)-vernolic acid	VOIL	QUA, FRA, FOR, COR, BIL, ALST, GAR
M284T713/M284T707	284.2225	11,89/11,78	C16H29NO3	2B	*N*-dodecanoyl-l-homoserine lactone		BIG
M127T51	127.0729	0.85	C3H6N6	2A	Melamine	FOR, JUST	FOR
M313T834	313.2741	13.89	C19H36O3	2B	(2E,18R)-18-hydroxynonadec-2-enoic acid	ARK	
M317T759/M317T743	317.2093	12.38/12.66	C20H28O3	2B	9-cis-4-hydroxyretinoic acid inumakoic acid, 15-deoxy-Delta(12,14)-prostaglandin (A2 or J2)	VOIL	QUA, FRA, GAR, TAS, FOR, ARK
M331T729	331.1885	12.16	C20H26O4	2B	(7Z)-lobohedleolide, hedychilactone D	VOIL	
M289T703	289.1781	11.97	C14H20N6O	4	Unidentified	VOIL	
M298T690	298.2382	11.51	C17H31NO3	4	Unidentified		BIG
M299T90	299.1191	1.49	C14H16F2N2O3	4	Unidentified	ARK	
M236T50	236.1496	0.83	C10H21NO5	4	Unidentified	VOIL	QUA, FRA
M371T92	371.1516	1.54	C17H24N4O2S	4	Unidentified	ARK, GAR	
M429T996	429.373	16.6	C29H50O3	2B	13-hydroxy-alpha-tocopherol	FRA, ARK, QUA, VOIL	GAR, ROC
M533T881	533.2548	14.68/14.87	C33H34N4O3	2B	Pyropheophorbide a	ARK, FRA, VOIL, QUA, JUST	FRA, ALST, SYT, EDF
M301T746	301.2156	12.38/12.43	C16H22O4	2A	Dibutyl phthalate		QUA, FRA
M419T933	419.316	15.34/15.55	C26H42O4	2A	Diisononyl phthalate	BIL	COR
M447T983	447.3471	16.17/16.38	C28H46O4	2A	Di-n-decyl phthalate, diisodecyl phthalate	QUA, BIL, FOR, VOIL	COR
M167T262	167.0342	4.36	C8H6O4	2A	Phthalic acid	ARK, FRA	

### Total organic carbon analysis and measurement

Total organic carbon (TOC) was performed on a Shimadzu TOC 5050 (Kyoto, Japan). From 16 to 54 µl of samples were injected in a vertical combustion tube hold at 680°C and half filled with platinum-based catalysis in a flow of 150 ml/min. Total carbon (TC) was converted into CO_2_ and measured on nondispersive infrared CO_2_ detector. Inorganic carbon (IC) was measured by the same amount of injection in a vessel containing phosphoric acid at a concentration of 25%. Carbon contained in carbonates was converted into CO_2_ and quantified on the same infrared detector. TOC concentration was obtained by the difference between TC and IC concentrations.

### Statistical and data analysis

Data analysis and statistical tests were carried out with R Statistical Software (v4.1.1, R Core Team 2018), using the *vegan* package for diversity analyses (Oksanen et al. 2018) and the *ggplot2* package for graphical representations (Wickham 2016). Heatmap graphs were plotted with the *complexHeatmap* package (Gu et al. 2016). Prokaryotic, fungal, and eukaryotic communities within-sample diversity also referred as alpha-diversity was estimated with the Shannon index (H’) while community dissimilarity also referred as beta diversity was estimated with the Bray–Curtis index. Shannon index was tested using either nonparametric tests (Kruskal–Wallis and paired Wilcoxon signed-rank tests with Benjamini–Hochberg correction) for bacteria or parametric tests (ANOVA and Tukey HSD *post hoc* tests) for fungi and other microeukaryotes. Beta diversity was analysed using permutational multivariate analysis (adonis2, R package *vegan*) (Legendre and Anderson 1999). Nonmetric multidimensional scaling (NMDS) plots using Bray–Curtis dissimilarity matrices were used to visualize beta diversity of microbial communities. Comparison of physicochemical parameters and organic molecule occurrence between NC and C was performed with partial least squares-discriminant analysis (PLS-DA) with *mixOmics*, R package (Rohart et al. 2017). The influence of the breeding site colonization status on the five most discriminant physicochemical parameters was tested by linear mixed model (lmm) after correcting for variations between interindividual garden effect. Considering organic molecules, data were not analysed with lmm because the residuals of the model were heteroskedasts and did follow a normal distribution. We therefore tested them using Kruskal–Wallis rank sum test, a nonparametric test. Principal coordinate analysis (PCA) were performed for NC and C samples independently in function of either physicochemical combined with pollution variables or organic molecule water occurrence. Monte Carlo test on the sum of the singular values of a procustean rotation was performed to test the concordance of each dataset. To analyse whether the abiotic factors of NC and C (either physicochemical and pollutant variables or organic molecule occurrence) were correlated with the microbial communities, we performed the same analysis. Then, we asked what was the proportion of the microbial communities within NC and C that could be explained by major variations in the abiotic factors. To that end, we have used the two first components (PC1_poll/PC2_poll or PC1_mp/PC2_mp, for physicochemical and pollution variables or organic molecules, respectively) of the abiotic factors’ PCA as an explanatory variable for a PERMANOVA analysis of the microbial communities Bray–Curtis dissimilarities. Then, we performed distance-based redundancy analyses (dbRDA) as constrained multivariate analyses to represent the microbial OTUs that were mostly influenced by PC1_poll/PC2_poll or PC1_mp/PC2_mp. Those OTUs were correlated with PC1_poll/PC2_poll or PC1_mp/PC2_mp using Spearman’s rank correlations.

Then, the objective was to develop a method allowing to determine relationships between variables of different natures, including categorical, numerical, and not following normal distributions, and then to visualize them with graph representations, in which nodes represent privileged relationships between those variables. To that end, we used a python code and visualized with the pyvis library. For each numerical variable (OTUs, Water T°, LC–HRMS data, and so on), we converted it into a binary value, such as exceptionally high values were identified as 1, and average or low values were identified as 0. Given the non-normal distribution of values, we used a robust variant of the z-score to identify exceptionally high values. More precisely, we computed for each variable the so-called modified z-score, computed using the median absolute deviation to the median instead of the standard deviation.

Each value x_i is thus transformed using the following formula:


\begin{eqnarray*}
\frac{{x\_i\ - med\left( x \right)}}{{MAD}},
\end{eqnarray*}

where “med(x)” corresponds to the median of variable “x” and “MAD” the median absolute deviation. We then defined a threshold above which a value is considered exceptional. In the second step, we used association rule scores to identify strong associations. More precisely we used Zhang’s score to assess how strongly related a variable is compared with another one (https://rasbt.github.io/mlxtend/user_guide/frequent_patterns/association_rules/). Zhang’s score is defined between −1 and +1, with 0 meaning no particular association. Here again, we defined a threshold to keep only values above a given threshold, i.e. having a strong enough association. Zhang’s score does not include statistical significance, so we add a statistical test (Binomial test, *P*-value < .05) *a posteriori* to assess the robustness of potential associations. An edge is added to the graph if the strength of the association is above the threshold and statistically significant. We used this method to avoid pitfalls with existing approaches.

## Results

### Noncolonized and colonized waters exhibit slight differences in physicochemical and organic molecule composition

**Figure 2.**
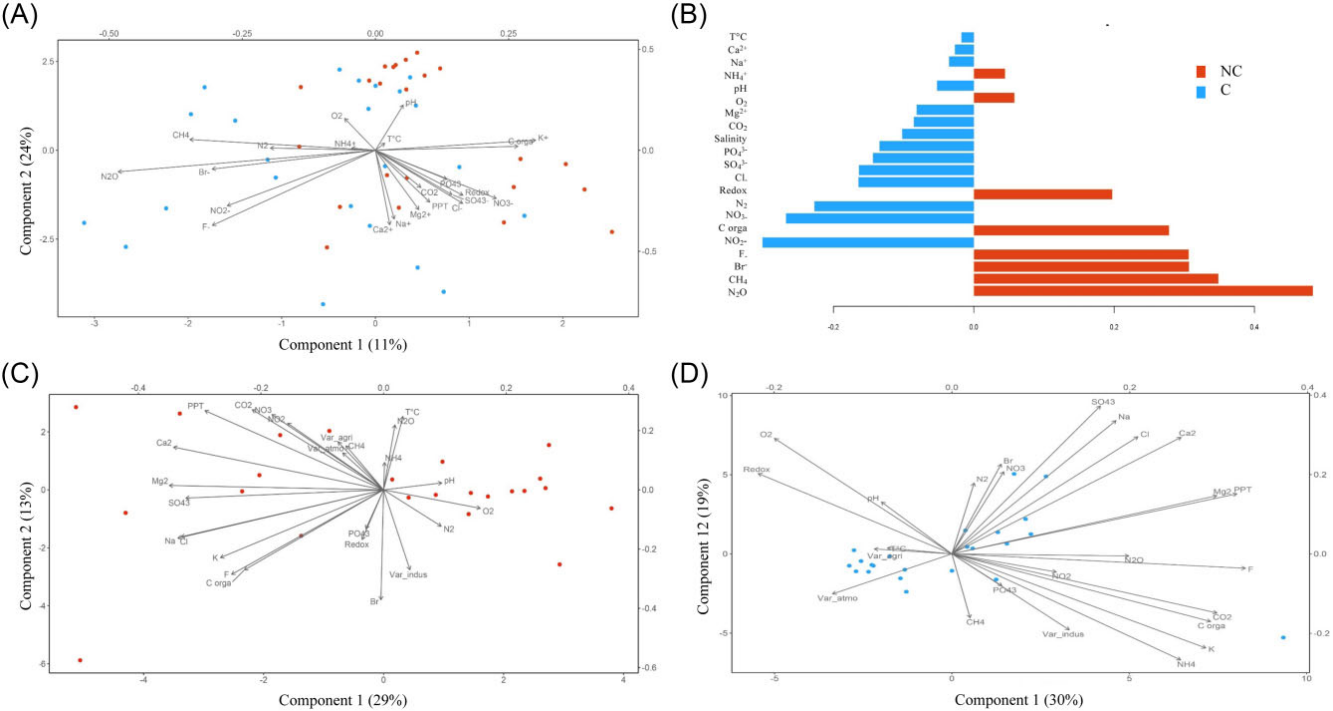
Variability of physicochemical parameters associated with noncolonized (NC) and colonized (C) water samples. (A) PLS-DA showing physicochemical parameters for NC (red circle) and C (blue circle). (B) Loading plot from the PLS-DA showing the physicochemical parameters with higher values in either the NC or C category for the first component of the PLS-DA (C) and (D) principal coordinate analysis (PCA) plots of the measured physicochemical parameters (black vectors) for NC (C) and C (D). “Var_indus,” “Var_agri,” and “Var_atmo” indicate calculated industrial, agricultural, and atmospheric pollution variables, respectively. “C Organ” corresponds to organic carbon content.

**Figure 3.**
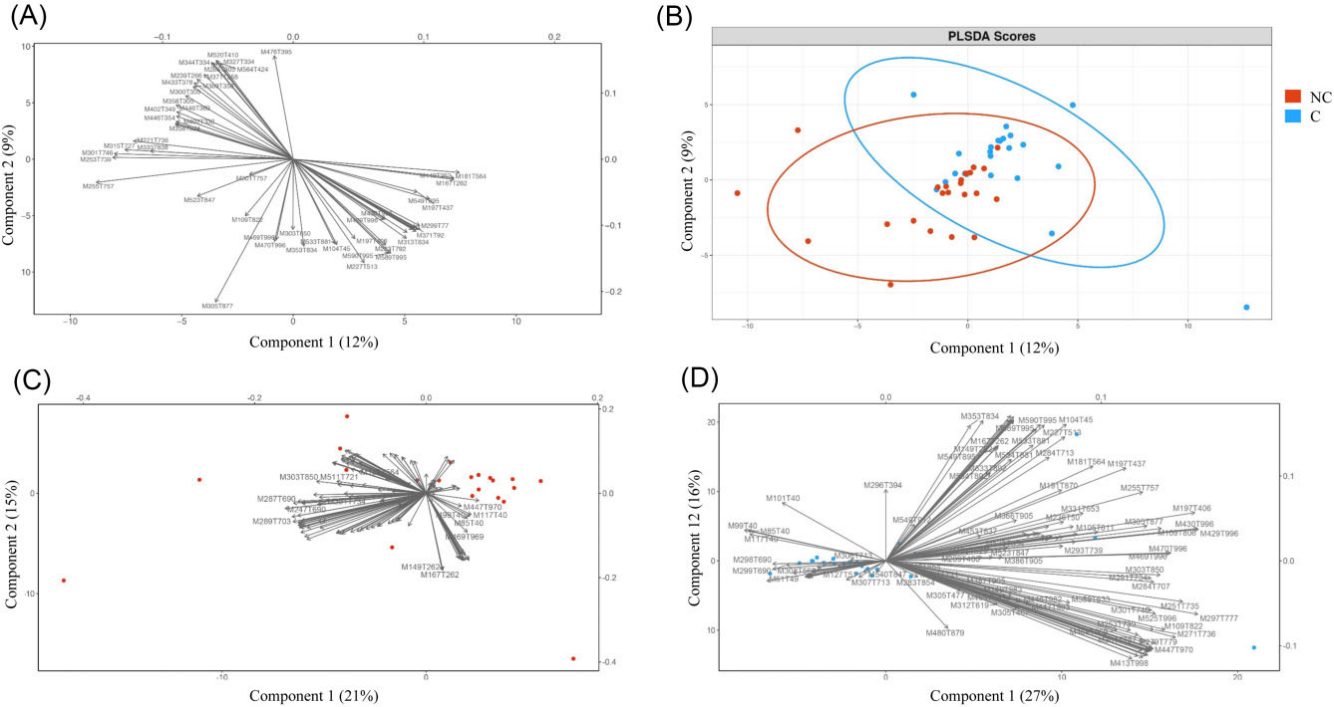
Variability of organic molecule occurrence associated with noncolonized (NC) and colonized (C) water samples. (A) Loading plot from the PLS-DA showing organic molecule variables most strongly correlated with latent variables. (B) Score plot showing organic molecule composition for NC (red circle) and C (blue circle). (C) and (D) PCA plots of organic molecules (black vectors) for NC (C) and C (D).

Following our selection criteria (see the section “Materials and methods”), a total of 23 gardens were selected for water sample collection. The characterization of each garden according to the level of atmospheric pollution, the calculated pollution variables and the distance from the garden to each pollution source is given in Table 1. The most common containers sampled were plastic rainwater collectors (87%), followed by plastic buckets (9%), and plastic watering cans (4%) (Table 3). Water physicochemical properties, i.e. ionic and gas composition, TOC, temperature, pH, conductivity, redox potential, and dissolved oxygen, are all summarized in Tables 3, 4a and 4b. We conducted a PLS-DA supervised analysis to identify the most discriminant physicochemical parameters between C and NC (Fig. 2A). Among the physicochemical properties, N_2_O and CH_4_ were significantly higher in NC than in C according to lmm (χ^2^ = 4.702, *P* = .03 and χ^2^ = 4.674, *P* = .03, respectively) (Fig. 2B). In fact, the average N_2_O and CH_4_ were two and nearly seven times higher in NC than in C, respectively. In a second analysis, NC and C samples were separated (Fig. 2C and D) to compare their physicochemical composition through a Procrustean analysis. The abiotic factors (physicochemical factors and pollution variables) had no significant effects on colonization status (NC or C) (Monte Carlo permutation test, observation = 0.345, *P* = .135). Regarding organic molecule analysis, the application of a nontargeted approach involving liquid chromatography coupled with quadrupole time-of-flight mass spectrometry (LC-QToF) allowed the detection of compounds that were found to be significantly different between C and NC from the same garden (*t*-test; *P* < .05) (Table S3). Among them, 22 features were putatively identified (Table 2). However, PLS-DA performed with all 147 features did not reveal significant differences between C and NC regarding organic molecule presence (*P* > .05) (Fig. 3A and B). When NC and C samples were separated (Fig. 3C and D) through the Procrustes analysis, no significant effect was found depending on the colonization status (NC or C) (Monte Carlo permutation test, observation = 0.06, *P* = .985).

**Table 3.** Characteristics of water samples and extemporaneous measures of physicochemical parameters. For each sample, a description of the colonization status, the type of water habitat, and measures of physicochemical properties are given. T(°C), salinity (PPT), pH, and 0_2_ dissolved (mg/l) are given for each sample individually and are also expressed per container as mean ± SE.

Sample ID	Garden	Sample type	Date	Type of sample	Matter	Color	T(°C)	Mean ± SE	Salinity	Mean ± SE	pH	Mean ± SE	0_2_ dissolved	Mean ± SE	Redox	Mean ± SE
ALST_NC	ALST	Noncolonized	22 July 2021	Rainwater collector	Plastic	Blue	25.17	21.5 (± 4)	0	0.15 (± 0.14)	8.49	7.4 (± 0.6)	3.48	6.4 (± 2)	49	198 (± 112)
ARK_NC	ARK		2 August 2021	Rainwater collector	Plastic	Blue	19.09		0		7.93		8.6		190	
AVIA_NC	AVIA		5 July 2021	Bucket	Plastic	Red	20.42		0		6.95		7.1		305	
BIG_NC	BIG		1 July 2021	Rainwater collector	Plastic	Blue	20.8		0		7.28		5.45		300	
BIL_NC	BIL		27 July 2021	Rainwater collector	Plastic	Blue	25.64		0.2		7.45		4.78		18	
BONN_NC	BONN		5 July 2021	Rainwater collector	Plastic	Green	22.18		0		7.27		7.46		283	
COR_NC	COR		22 September 2021	Rainwater collector	Plastic	White	14.95		0.3		7.35		8.51		250	
DEC_NC	DEC		16 June 2021	Rainwater collector	Plastic	Blue	28.09		0.3		7.29		6.74		265	
EDF_NC	EDF		20 July 2021	Rainwater collector	Plastic	Blue	26.38		0		7.99		2.64		105	
ESP_NC	ESP		8 July 2021	Rainwater collector	Plastic	Blue	20.13		0		8.28		6.91		188	
FOR_NC	FOR		19 July 2021	Watering can	Plastic	White	21.3		0		6.15		6.6		285	
FRA_NC	FRA		29 September 2021	Rainwater collector	Plastic	Blue	17.7		0		6.98		7.86		245	
GAR_NC	GAR		31 August 2021	Rainwater collector	Plastic	Blue	17.7		0		7.98		9		206	
JUST_NC	JUST		25 June 2021	Rainwater collector	Plastic	Blue	20.23		0.1		7.59		3.28		70	
MID_NC	MID		3 August 2021	Rainwater collector	Plastic	Black	19.2		0.4		7.04		12.25		271	
MOU_NC	MOU		26 July 2021	Rainwater collector	Plastic	Gray	23.02		0		6.41		7.94		214	
PER_NC	PER		7 September 2021	Rainwater collector	Plastic	Blue	21.8		0.1		7.64		3		257	
QUA_NC	QUA		6 September 2021	Rainwater collector	Plastic	White	24.93		0.1		7.44		6.56		192	
REB_NC	REB		1 September 2021	Rainwater collector	Plastic	Blue	20.72		0.1		6.38		6.14		288	
RECU_NC	RECU		13 September 2021	Bucket	Plastic	White	17.01		0.2		6.58		6.42		254	
SYT_NC	SYT		21 July 2021	Rainwater collector	Metal	Gray	24.94		0.1		7.46		3.91		−158	
TAS_NC	TAS		23 September 2021	Bucket	Plastic	Black	14.82		0.1		8.17		5.96		228	
VOIL_NC	VOIL		17 June 2021	Rainwater collector	Plastic	Blue	29.12		0.2		8.05		5.83		245	
ALST_C	ALST	Colonized	22 July 2021	Rainwater collector	Plastic	Blue	25.51	21.6 (± 3)	0.3	0.19 (± 0.19)	8.19	7.4 (± 0.8)	5.04	6.5 (± 2)	230	215 (± 88)
ARK_C	ARK		2 August 2021	Rainwater collector	Plastic	Blue	22.25		0.4		6.72		3.12		−64	
AVIA_C	AVIA		5 July 2021	Rainwater collector	Plastic	Green	20.92		0		7.95		7.17		252	
BIG_C	BIG		1 July 2021	Rainwater collector	Plastic	Blue	21.98		0		8.02		8.85		317	
BIL_C	BIL		27 July 2021	Rainwater collector	Plastic	Blue	23.4		0		8.05		3.21		86	
BONN_C	BONN		5 July 2021	Rainwater collector	Plastic	Blue	21.22		0		7.3		6.79		199	
COR_C	COR		22 September 2021	Rainwater collector	Plastic	Blue	15.97		0.3		6.7		8.64		231	
DEC_C	DEC		16 June 2022	Rainwater collector	Plastic	Blue	26.29		0.012		7.63		10.12		194	
EDF_C	EDF		20 July 2021	Rainwater collector	Plastic	Blue	23.81		0		7.8		5.02		248	
ESP_C	ESP		8 July 2021	Rainwater collector	Plastic	Blue	20.29		0		7.99		7.39		165	
FOR_C	FOR		19 July 2021	Rainwater collector	Plastic	Gray	23.71		0		7.85		5.75		296	
FRA_C	FRA		29 September 2021	Watering can	Plastic	Green	18.13		0.1		5.7		10.07		264	
GAR_C	GAR		31 August 2021	Rainwater collector	Plastic	Blue	18.45		0.3		7.97		6.82		160	
JUST_C	JUST		25 June 2021	Rainwater collector	Plastic	Blue	20.83		0.1		7.67		6.91		260	
MID_C	MID		3 August 2021	Rainwater collector	Plastic	Blue	20.43		0		7.28		4.56		284	
MOU_C	MOU		26 July 2021	Rainwater collector	Plastic	Blue	23.68		0.3		7.79		7.02		237	
PER_C	PER		7 September 2021	Rainwater collector	Plastic	Blue	21.5		0.1		7.26		1.95		71	
QUA_C	QUA		6 September 2021	Rainwater collector	Plastic	Blue	21.86		0		7.17		5.09		254	
REB_C	REB		1 September 2021	Rainwater collector	Plastic	Blue	21.21		0		5.72		8.32		328	
RECU_C	RECU		13 September 2021	Rainwater collector	Plastic	Blue	19.91		0.2		6.12		5.03		277	
SYT_C	SYT		21 July 2021	Rainwater collector	Metal	Gray	23.24		0.2		7.63		4.11		207	
TAS_C	TAS		23 September 2021	Bucket	Plastic	White	16.21		0.2		7.78		7.55		256	
VOIL_C	VOIL		17 June 2021	Rainwater collector	Plastic	White	25.97		0		8.76		9.23		199	

**Table 4a.** Measures of water ionic composition in noncolonized and colonized water samples. The concentrations of each ion (F^−^, Cl^−^, Br^−^, NO_2_^−^ NO_3_^−^, PO4_3_^−^, SO4_3_^−^, Na^+^, NH_4_^+^, K^+^, Mg^2+^, and Ca_2_^+^) are given for each sample individually and are also expressed per container as mean ± SE.

		Water ionic composition																							
Sample ID	Sample type	F^−^	Mean ± SE	Cl^−^	Mean ± SE	NO_2_^−^	Mean ± SE	Br^−^	Mean ± SE	NO_3_^−^	Mean ± SE	PO_4_^3−^	Mean ± SE	SO_4_^3−^	Mean ± SE	Na^+^	Mean ± SE	NH_4_^+^	Mean ± SE	K^+^	Mean ± SE	Mg^2+^	Mean ± SE	Ca^2+^	Mean ± SE
ALST_NC	NC	0.01	0.06 (+/− 0.06)	0.32	8.1 (+/− 11)	0.02	0.04 (+/− 0.02)	0.03	0.3 (+/− 0.7)	0.13	1.2 (+/− 2.5)	0	0.5 (+/− 0.4)	1.28	10.7 (+/− 12)	0.85	5.9 (+/− 7)	0.57	0.34 (+/− 0.4)	0.61	3.2 (+/− 3.2)	0.24	3.1 (+/− 3.4)	4.87	25 (+/− 21)
ARK_NC		0.03		0.22		0		2.6		0.01		0.07		0.46		1.2		0.02		0.58		0.15		2.92	
AVIA_NC		0.03		1.58		0.04		0.36		0.66		0.81		3.82		1.78		0.47		3.41		0.92		11.85	
BIG_NC		0.02		0.54		0.02		0.04		0.22		0.13		0.92		1.56		0.81		0.77		0.26		3.83	
BIL_NC		0.07		8.56		0.07		0.04		0.54		0.51		17.19		6.76		0		4.87		4.97		44.9	
BONN_NC		0.04		0.38		0.01		0.01		0.02		0		0.88		0.8		0.03		0.39		0.24		5.29	
COR_NC		0.11		31.27		0.05		0.1		2.24		0.36		33.15		20.66		0.41		5.52		6.3		62.76	
DEC_NC		0.04		25.65		0.02		0.02		10.33		0		18.31		15.21		0.02		3		6.21		65.03	
EDF_NC		0.02		0.28		0.01		0.13		0.21		0.07		0.58		0.41		0.29		0.55		0.22		4.38	
ESP_NC		0.02		0.18		0.03		0.01		0.13		0		0.43		0.53		0.02		0.12		0.4		9.57	
FOR_NC		0.02		0.82		0.02		0.1		0.48		0.63		1.32		1.75		0.09		1.49		0.38		4.55	
FRA_NC		0.24		42.46		0		1.86		0.02		0.64		22.82		26.91		0.4		14.85		8.46		38.82	
GAR_NC		0.03		0.73		0.03		0.03		1.83		1.61		2.38		1.26		0.24		6.1		1.16		10.42	
JUST_NC		0.09		1.23		0.05		0.4		0.79		0.3		2.82		1.55		0.93		4.38		0.62		16.65	
MID_NC		0.05		20.3		0.08		0		6.52		0.09		26.5		13.62		0.16		5.72		11.55		71.5	
MOU_NC		0.01		0.22		0.01		0.04		0.03		0		0.76		0.31		0		0.27		0.12		5.61	
PER_NC		0.04		2.04		0.05		0		0.56		0.52		2.9		1.37		0.03		3.01		0.68		18.66	
QUA_NC		0.18		12.09		0.03		0		0.02		0.06		33.61		8.72		0		2.93		6.59		24.46	
REB_NC		0.07		4.21		0.05		0.01		2.78		0.52		11.18		3.28		0.02		1.94		2.08		25.12	
RECU_NC		0.11		11.41		0.07		0		0.1		0		16.36		9.72		0.15		2.08		7.41		38.73	
SYT_NC		0.1		7.33		0.02		0.01		0.02		0		8.83		5.54		1.7		4.13		4.26		29.31	
TAS_NC		0.06		3.15		0.08		0.21		0.33		0		9.36		3.43		0.43		1.02		2.13		23.81	
VOIL_NC		0.07		11.08		0.05		0		0.45		0.87		30.65		7.8		0.04		6.04		5.06		48.95	
ALST_C	C	0.08	0.05 (+/− 0.3)	17.52	10.4 (+/− 15)	0.01	0.04 (+/− 0.02)	1.35	0.13 (+/− 0.3)	4.12	1.86 (+/− 3)	0	0.48 (+/− 0.8)	34.32	13 (+/− 17)	13.2	6.1 (+/− 7.4)	0.05	0.5 (+/− 0.8)	3.4	9.1 (+/− 26)	7.81	3.4 (+/− 3.7)	64.8	26 (+/− 24)
ARK_C		0.16		16.48		0.03		0		0.05		0.93		3.26		6.19		3.09		125.24		11.18		39.99	
AVIA_C		0.02		0.34		0.01		0.09		0.69		0.15		1.48		1		0.26		0.58		0.23		2.99	
BIG_C		0.02		0.48		0.01		0.02		0.57		0.15		0.87		1.44		0		0.46		0.18		3.33	
BIL_C		0.03		0.53		0.02		0.02		0.31		0.06		1.21		0.99		0.84		0.7		0.23		3.86	
BONN_C		0.02		1.17		0.03		0.01		0.82		0.55		2.49		0.77		0.02		2.66		0.91		9.95	
COR_C		0.06		33.53		0.07		0		3.16		0		34.2		19.89		0		2.47		5.99		45.37	
DEC_C		0.02		0.62		0.02		0.01		0.48		0.1		0.98		1.82		0.09		0.46		0.16		4.44	
EDF_C		0.04		18.65		0.02		0		6		0.01		27.6		13.24		0		2.4		10.28		45.43	
ESP_C		0.02		4.16		0.02		0.04		2.04		0.19		4.61		1.96		0		1.01		0.73		14.45	
FOR_C		0.01		0.23		0.01		0		0.4		0.09		0.79		0.86		0		0.19		0.12		2.46	
FRA_C		0.11		3.32		0.07		0.01		0.08		0.24		3.94		1.01		0.39		21.46		3.97		26.81	
GAR_C		0.05		29.23		0.05		0.04		3.6		0.79		28.99		5.51		0.12		8.26		4.33		72.61	
JUST_C		0.05		4.21		0.06		0.02		12.04		3.41		12.95		6.86		0		16.66		2.37		32.71	
MID_C		0.02		0.25		0.01		0.05		0.64		0.06		1.24		0.74		0.08		0.32		0.2		5.63	
MOU_C		0.07		62.57		0		0		3.32		0.07		65.65		29.4		0		1.82		8.23		75.23	
PER_C		0.07		0.59		0.07		0.02		0.12		0.45		0.92		1.08		0.09		2.49		0.83		19.95	
QUA_C		0.02		0.37		0.02		0.01		0.82		1.15		0.98		0.42		0.18		5.14		0.35		2.65	
REB_C		0.02		0.41		0.01		0.01		0.7		0.24		0.63		0.68		0		0.24		0.11		1.89	
RECU_C		0.06		13.21		0.07		0		0.45		0.13		9.26		9.8		0		3.01		8.44		34.69	
SYT_C		0.07		19.25		0.03		0.02		1.73		0		29.7		13.3		0		6.2		4.03		37.31	
TAS_C		0.06		10.81		0.04		0.52		0.5		0		30.44		8.8		0.91		3.31		6.36		34.97	
VOIL_C		0.04		1.42		0.02		0.01		0.07		0.33		1.16		2.29		0.11		0.86		0.34		5.27	

**Table 4b.** Measures of gas composition as well as TOC in noncolonized and colonized water samples. The concentrations of each gaz (O_2_, N_2_, CH_4_, CO_2_, and N_2_O) as well as TOC are given for each sample individually and are also expressed per container as mean ± SE.

		Gas composition of water											
Sample ID	Sample type	O_2_ (%)	Mean ± SE	N_2_ (%)	Mean ± SE	CH_4_ (ppm vol)	Mean ± SE	CO_2_ (ppm vol)	Mean ± SE	N_2_O (ppm vol)	Mean ± SE	TOC	Mean ± SE
ALST_NC	NC	2.11	2.2 (+/− 1)	5.02	5.7 (+/− 3)	18.74	14 (+/− 43)	2373.8	3559 (+/− 3592)	3.67	1.1 (+/− 1.3)	4.37	10 (+/− 9.9)
ARK_NC		2.08		4.65		0.17		770.9		0.14		1	
AVIA_NC		2.16		5.12		0.25		2071.3		2.74		7.9	
BIG_NC		2.12		5.02		0.18		2065.25		0.5		5.77	
BIL_NC		1.83		4.29		198.23		4200.24		0.73		11.61	
BONN_NC		1.87		4.42		0.04		5512.88		0.02		6.84	
COR_NC		1.77		5.55		0.24		2674.62		1.49		24.92	
DEC_NC		2.12		4.97		0.12		11662.99		1.42		7.8	
EDF_NC		2.01		4.35		1.01		2098.92		1.02		6.56	
ESP_NC		6.45		21.22		0.37		365.14		0.49		3.18	
FOR_NC		2.07		5.22		0.14		1904.64		1.12		12.72	
FRA_NC		1.58		7.44		15.9		4674.8		0.37		39.45	
GAR_NC		2.85		6.43		0.22		1947.26		0.64		12.58	
JUST_NC		2.09		4.57		11.23		2212.85		4.79		9.09	
MID_NC		1.6		4.01		0.18		12075.54		1.6		5.5	
MOU_NC		2.28		5.2		0.11		33.86		0.55		3.98	
PER_NC		2.12		4.8		0.18		1978.87		0		22.82	
QUA_NC		1.97		4.65		0.17		417.37		0.28		14.52	
REB_NC		2.19		4.61		0.23		1358.77		0.01		5.12	
RECU_NC		2.2		5.15		1.24		3593.56		0.24		28.09	
SYT_NC		0.69		4.67		69.23		12225.67		2.76		16.5	
TAS_NC		1.95		5.56		0.36		2383.38		1.51		20.04	
VOIL_NC		2.11		5.08		1.52		3248.9		0.05		17.2	
ALST_C	C	3.67	2.1 (+/− 0.6)	10.83	5.2 (+/− 1.3)	0.46	2.4 (+/− 8.5)	8966.9	3986 (+/− 5709)	1.67	0.6 (+/− 0.8)	7.65	10.6 (+/− 8.4)
ARK_C		0.31		5.22		9.04		28552.01		2.22		66	
AVIA_C		2.15		4.7		0.04		2038.17		0.2		3.2	
BIG_C		3		6.58		0.24		1192.21		0.22		2.51	
BIL_C		1.97		4.7		40.54		4233.84		0.54		6.33	
BONN_C		2.35		5.33		0.06		1735.24		0.13		31.65	
COR_C		2.1		4.41		0.01		1809.48		0.05		32.97	
DEC_C		2.04		5.07		0.95		2967.18		0.02		3.05	
EDF_C		1.96		4.1		0.24		4611.83		0.05		3.77	
ESP_C		2.42		5.23		0.06		789.28		0.51		5.2	
FOR_C		2.2		4.93		0.23		1577.37		0.26		4.25	
FRA_C		1.91		5.08		0.14		1516.77		0.02		35.76	
GAR_C		1.78		4.51		0.25		6583.94		0.82		29.63	
JUST_C		1.91		4.68		0.12		3391.08		0.59		17.4	
MID_C		2.46		5.55		0.24		1694.02		0.74		3	
MOU_C		2.24		4.96		0.02		4190.09		0.06		7.84	
PER_C		2.34		5.71		1.48		4109.99		0.7		22.06	
QUA_C		2.12		4.76		0.3		2264.26		0.48		11.97	
REB_C		2.11		4.37		0.04		731.88		0.01		2.14	
RECU_C		2.07		4.88		0.5		3726.21		0.48		21.39	
SYT_C		2.11		4.81		0.18		977.73		3.01		15.12	
TAS_C		1.95		4.4		0.21		2757.52		0.77		24.15	
VOIL_C		1.91		4.66		0.5		1270.64		0.01		14.1	

### Fungal composition differs between noncolonized and colonized waters while microbial community structure is similar between larvae and water samples, regardless of colonization status

**Figure 4.**
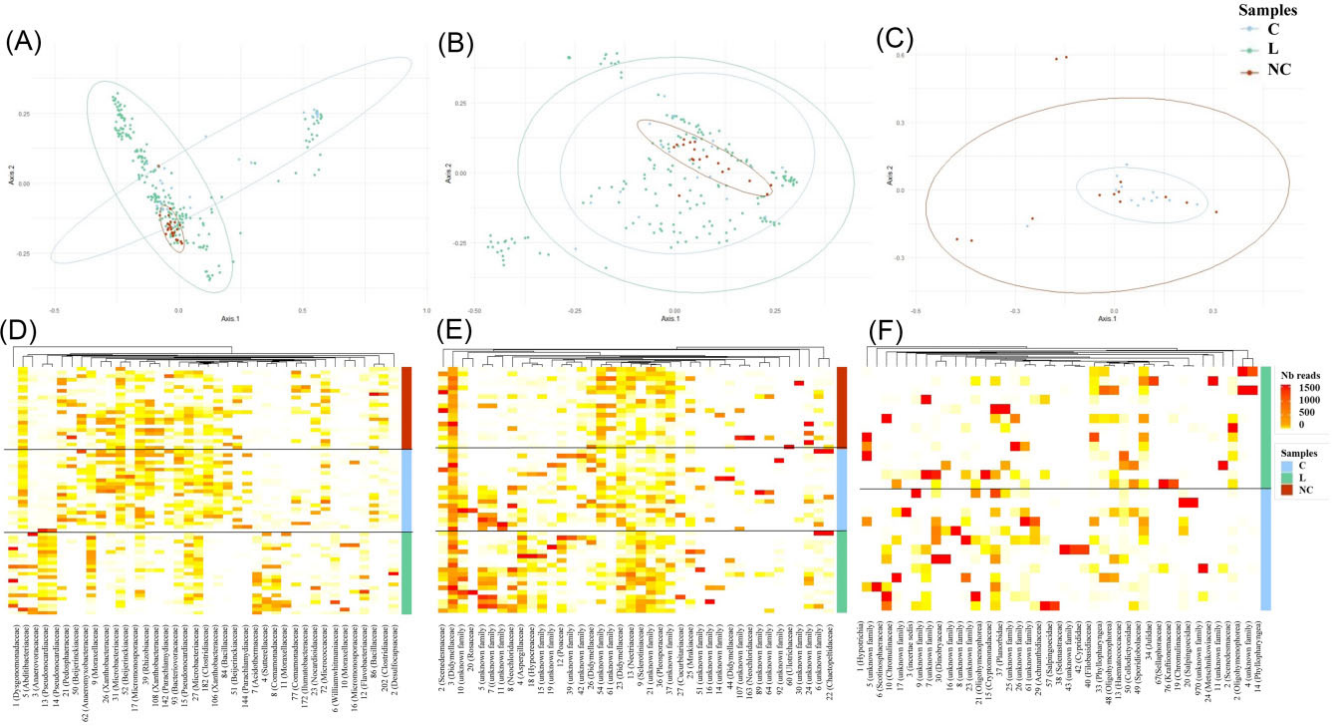
Microbial community composition and structure associated with noncolonized waters (NC), colonized waters (C) and larvae (L). NMDS ordinations displaying OTU composition across NC, C, and L are shown for each category of microorganisms: (A) bacteria, (B) fungi, and (C) microeukaryotes. Heatmaps with cluster categorization representing the number of reads of the 40 most abundant OTUs among the total (D) bacterial, (E) fungal, and (F) microeukaryotic OTUs (columns) for each type of sample [noncolonized, colonized, larvae (average of 10 larvae)] (rows). The intensity of the color corresponds to the relative abundance of OTUs for each sample.

A total of 1373 bacterial OTUs belonging to 27 phyla and 329 genera were identified from the 23 gardens (23 NC, 23 C, and their associated larvae). NC and C exhibited similar bacterial compositions at the phylum level (Fig. S1A). The predominant phylum was Proteobacteria (37% and 34%), followed by Actinobacteriota (20% and 24%) and Firmicutes (17% and 12%) for NC and C, respectively (Fig. S1A). In larvae, the most abundant bacterial phylum was Proteobacteria (35%), followed by Actinobacteriota (24%) and Bacteroidota (17%) (Fig. S1A). At the genus level, 40%, 34%, and 31% of the bacterial genera displayed low abundance (<1%) in NC, C, and L, respectively (Fig. S1B). Among the 42 most abundant genera, 7 were common (e.g. *Rhizobium, Legionella*, and *Abditibacterium*), and others were specific to NC (6), C (7), and larvae (14) (Fig. S1B). Interestingly, *Actinomycetospora* and *Actinoplanes* were abundant in C and L but in low abundance in NC (<1%). The alpha diversities of the bacterial communities in NC and C were comparable (Wilcoxon pairwise; *P* = .53), while that in L was significantly lower than that in C (Wilcoxon pairwise; *P* = .0012) and NC (Wilcoxon pairwise; *P* = .0005) (Table S4). Regarding the fungal community (i.e. ITS sequencing), a total of 18 gardens were analysed out of the 23 sampled gardens (with 18 NC, 18 C, and their associated larvae). If the ITS sequencing preferentially targeted and revealed the presence of fungi (56.4%, 56.6%, and 57.8%), other eukaryotic kingdoms such as Viridiplantae were also detected (15.4%, 16.4%, and 15.8%) for NC, C, and L, respectively, followed by three minority kingdoms (Rhizaria, Protista, and Ichthyosporia) (data not shown). Regarding fungal communities, a total of 716 fungal OTUs belonging to 8 phyla and 264 genera were identified. *Ascomycota* was the predominant phylum (47%, 55%, and 72% in NC, C, and L, respectively), followed by Basidiomycota (27%, 24%, and 16%) (Fig. S2A). The phylum Rozellomycota was the third most abundant phylum in NC and C (7% and 6%) but represented only 0.5% of the total fungi in L (Fig. S2A). At the genus level, 40%, 34%, and 31% of the fungal genera displayed a low abundance (<1%) in NC, C and L, respectively (Fig. S2B). Only *Dioszegia* was common to NC and C, while *Phoma* was shared among NC, C, and L (Fig. S2B). Interestingly, *Dacrymyces* and *Pyrenochaetopsis* were abundant in C and L but very low in abundance in NC (0.18% and 0.04%, respectively) (Fig. S2C). Comparison analysis performed with the Shannon index indicated no significant differences in the alpha diversity of fungal communities between NC and C (Tukey HSD *post hoc* tests, t ratio = 0.559, df = 51, *P* = .84) or between C and L (Tukey HSD *post hoc* tests, t ratio = −1.914, df = 51, *P* = .14) (Table S4). Conversely, the alpha diversity of fungal communities was significantly higher in L than in NC (Tukey HSD *post hoc* test, t ratio = 2.473, df = 51, *P* = .04) (Table S4). Regarding eukaryotic communities, a total of 739 eukaryotic OTUs belonging to 38 phyla and 181 genera were identified among 13 gardens (13 NC and 13 C). In contrast to bacteria and fungi, the main eukaryotic phyla differed between NC and C. The predominant phylum was Ciliophora (22.5%), followed by Chlorophyta (18.4%) and Ochrophyta (10.2%), in NC, while the predominant phylum was Bicosoecida (19.5%), followed by Ochrophyta (14.6%) and Chlorophyta (9.8%), in C (Fig. S3A). At the genus level, 10.3% and 14.9% of the eukaryotic genera displayed low abundance (<1%) in NC and C, respectively (Fig. S3B). Among the 28 most abundant genera, three were common (*Sterkiella, Scotinosphaera*, and *Goniomonas*), and others were specific to NC (13) and C (11) (Fig. S3B). The alpha diversities of the eukaryotic communities in NC and C were comparable (Tukey HSD *post hoc* test, *P* = .9) (Table S4). NMDS based on Bray‒Curtis dissimilarity revealed no differences in microbial composition between NC and C (*adonis2*-PERMANOVA, *R*^2^ = 0.02, *P* = .77 for bacteria; *R*^2^ = 0.04, *P* = .7 for eukaryotes) (Fig. 4A–C). However, significant differences in fungal composition were found between NC and C (*adonis2*-PERMANOVA, *R*^2^ = 0.04, *P* = .03). Moreover, significant differences in bacterial composition were observed between NC and L (*adonis2*-PERMANOVA, *R*^2^ = 0.03, *P* = .001) as well as between C and L (*adonis2*-PERMANOVA, *R*^2^ = 0.03, *P =* .001). Heatmaps showed contrasting patterns with some OTUs unique (specific) to certain categories of samples or shared (OTUs that are common among several categories of samples) (Fig. 4D–F). Among fungi, OTU 3 (family *Didymellaceae*) was present in all samples. Conversely, the bacterial OTUs 50, 51, and 52 (family Beijerinckiaceae), 62 (family Anaeromyxobacteraceae), 84 (family Bacillaceae), 144 (family Parachlamydiaceae), and 202 (family Clostridiaceae) as well as the fungal OTU 54 (not identified) were found in substantial proportions in NC and C but absent from L. Two other bacterial OTUs (OTU 13 and OTU 14) belonging to family Pseudonocardiaceae were enriched in L compared to C.

### Organic molecules and physicochemical properties differentially affect microbial communities in noncolonized and colonized waters

**Figure 5.**
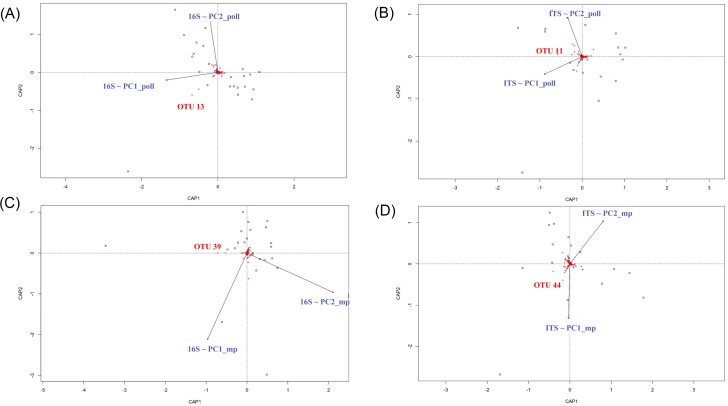
Effect of pollution gradients on the structure and variability of microbial communities associated with colonized (C) water samples. Microbial community distances among samples (dots) were constrained based on physicochemical and pollution parameters (blue vectors) and are represented by dbRDA plots. PC1_poll/PC2_poll represents the combination of physicochemical and pollution variables, and PC1_mp/PC2_mp represents the organic molecule occurrence. OTU names significantly correlated (*P* < .05) with PC1_poll/PC2_poll or PC1_mp/PC2_mp are highlighted in red. Only significant effects are shown. (A) and (B) PC1_poll/PC2_poll dbRDA was performed for microbial community distances obtained from 16S rRNA and ITS sequencing, respectively. (C) and (D) PC1_mp/PC2_mp dbRDA was performed for microbial community distances obtained from 16S rRNA and ITS sequencing, respectively.

**Figure 6.**
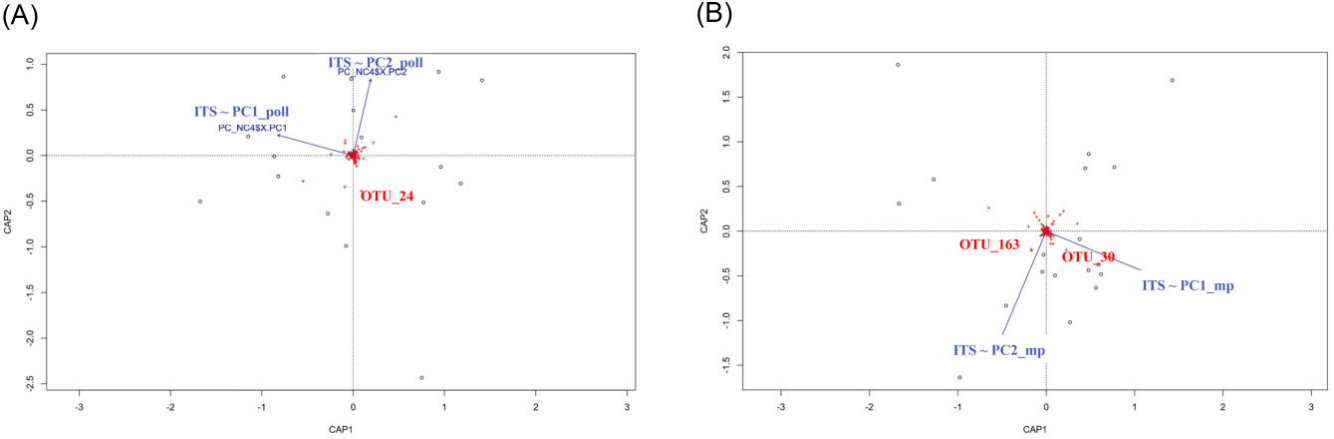
Effect of pollution gradients on the structure and variability of microbial communities associated with noncolonized (NC) water samples. Microbial community distances obtained from ITS sequencing (dots) were constrained based on physicochemical and pollution parameters (blue vectors) and represented by dbRDA plots. (A) PC1_poll/PC2_poll represents the combination of physicochemical and pollution variables. (B) PC1_mp/PC2_mp represents the organic molecule occurrence. OTU names significantly correlated (*P* < .05) with PC1_poll/PC2_poll or PC1_mp/PC2_mp are highlighted in red.

According to previous analyses, coordinates of the two first components of PCA (PC1_poll and PC2_poll from Fig. 2C and D and PC1_mp and PC2_mp from Fig. 3C and D) were extracted and subsequently used to reflect the distribution of samples according to either physicochemical and pollution variables or the occurrence of organic molecules in terms their microbial composition. We then evaluated whether those variables shape the microbial structure of NC and C. For this purpose, db-RDAs were conducted to maximize the separation of water microbial communities based on differences in either their physicochemical composition and pollution context (PC1_poll and PC2_poll) or organic molecule structure (PC1_mp and PC2_mp) (Figs 5 and 6). In colonized water samples, the structure of bacterial communities was significantly correlated with PC1_poll (*adonis2*-PERMANOVA, *R*^2^ 0.07 = 0.03, *P* = .001), PC2_poll (*adonis2*-PERMANOVA, *R*^2^ = 0.06, *P* = .015) and PC2_mp (*adonis2*-PERMANOVA, *R*^2^ = 0.44, *P* = .007), while that of fungal communities varied only according to PC1_poll (*adonis2*-PERMANOVA, *R*^2^ = 0.07, *P* = .006). The most important microbial OTUs involved in these separations were bacterial OTU 13 (identified as *Actinomycetospora*) and fungal OTU 11 (unidentified Rozellomycota), which correlated with high values of PC1_poll (*P* = .017, Rhô = 0.5 for OTU 13 and *P* = .003, Rhô = 0.66 for OTU 11), as well as bacterial OTU 39 (identified as Rhizobiaceae) (Fig. 5A and B) and fungal OTU 44 (identified as *Ascochyta phacae*), which correlated with low and high values of PC2_mp, respectively (*P* = .008, Rhô = 0.54 for OTU 39 and *P* = .04, Rhô = 0.48 for OTU 44) (Fig. 5C and D). Regarding noncolonized water samples, the structure of microbial communities was significantly influenced by PC1_poll (*adonis2*-PERMANOVA, *R*^2^ = 0.06, *P* = .027 for bacteria and *adonis2*-PERMANOVA, *R*^2^ = 0.1, *P* = .04 for eukaryotes) as well as by PC1_mp (*adonis2*-PERMANOVA, *R*^2^ = 0.08, *P* = .025 for fungi). The most important clusters involved in these separations were OTU 24 (identified as *Haematococcus*), which correlated with PC1_poll (*P* = .017, Rhô = 0.6) (Fig. 6A), and OTU 30 (unidentified Rozellomycota) and OTU 163 (identified as *Neochloris aquatica*), which correlated with PC1_mp (*P* = .03, Rhô = 0.5 and *P* = .04, Rhô = 0.48 for OTU 30 and OTU 163, respectively) (Fig. 6B).

### Organic molecules and microbial communities are highly connected in colonized water samples

**Figure 7.**
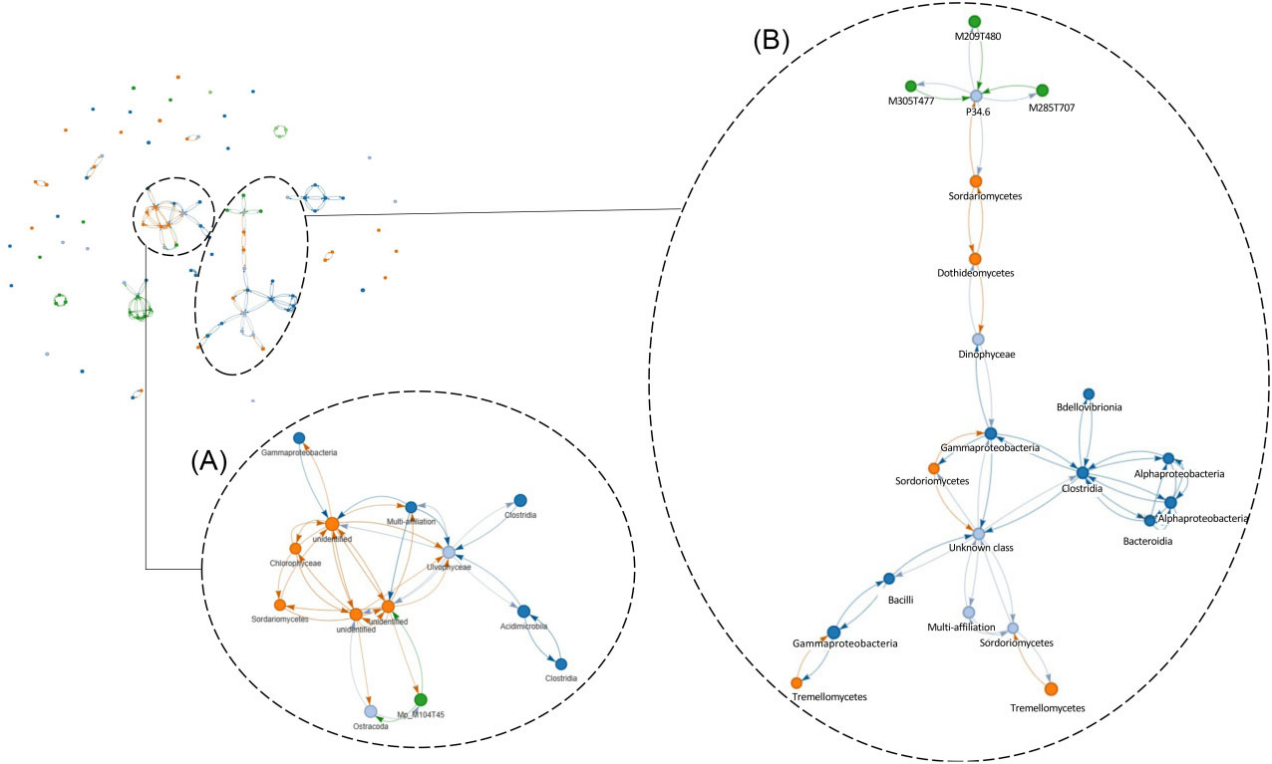
A network analysis showing positive correlations between microbial OTUs, i.e. bacteria (dark blue dots), fungi (orange dots), and eukaryotes (light blue dots), organic molecules (green dots), and physicochemical parameters (light green dots). Each link represents a correlation with ρ > 0.25. For this analysis, we kept the samples with positive PCR amplification for each genetic marker, for a total of 13 colonized waters among the 23 sampled.

A network analysis was performed on colonized water samples to explore associations between microbial OTUs (i.e. bacterial, fungal, and eukaryotic OTUs), physicochemical parameters, pollution variables, and organic molecules (Fig. 7). Two independent networks of positive correlations that contained interkingdoms associations and organic molecules were characterized (Fig. 7). In the first network, microbial co-occurrence patterns included the eukaryotic OTU 9 (identified as Ostracoda), five bacterial OTUs (i.e. OTU 77 identified as the genus *Azohydromonas*, OTU 355 identified as the class Acidimicrobiia, unidentified OTU 630, and OTUs 186 and 659 identified as the class Clostridia), five fungal OTUs (i.e. OTU 8 identified as the species *N. aquatica*, OTU 188 identified as the species *Humicola grisea* and unidentified OTUs 5, 7, and 105) and one unidentified feature (M104T45) (Fig. 7A). The second network showed module associations between nine bacterial OTUs (i.e. 89, 93, 112, 130, 275, 590, 684, and 1018 identified as belonging to the genera *Bacillus, Bacteriovorax, Brevundimonas, Candidatus Methylopumilus*, and *Flavobacterium*, the families *Yersiniaceae* and Rhizobiaceae and the genus *Tyzzerella*, respectively), seven fungal OTUs (i.e. OTUs 37, 68, 114, 127, 132, and 298 identified as belonging to the order Pleosporales and the genera *Coniochaeta, Vishniacozyma, Filobasidium, Coniochaeta*, and *Coniochaeta*, respectively), eight eukaryotic OTUs (i.e. OTUs 7, 8, and 736 identified as belonging to the phylum Bicosoecida and OTUs 68, 94, 125, 265, and 423 identified as belonging to the class Klebsormidiophyceae, the genus *Coniochaeta*, the phylum Ascomycota, the genus *Gyrodinium* and the class Chytridiomycetes), and three unidentified features (M305T477, M209T480, and M285T707). In this second network, we focused on the eukaryotic OTU 68 that was associated with two eukaryotic OTUs (i.e. OTU 94 identified as belonging to the class Sordariomycetes and OTU 125 to the class Chrytridiomycetes), the fungal OTU 132 identified as Sordariomycetes and three bacterial OTUs associated with three different classes (OTU 89 identified as Bacilli, OTU 590 identified as Gammaproteobacteria, and OTU 1018 identified as Clostridia) (Fig. 7B). Finally, the eukaryotic OTU 7 belonging to the phylum Bicosoecida was associated with three features (M305T477, M209T480, and M285T707).

## Discussion

Whether biotic and abiotic factors directly or indirectly impact the ability of mosquitoes to thrive in aquatic habitats remains poorly investigated. We addressed this question by specifically studying *Ae. albopictus*, one of the most invasive mosquito species in the world. As the type of container was shown to influence water physicochemical parameters (Hery et al. 2021), our analysis mainly focused on rainwater collectors. Rainwater harvesting is a widely used method to collect and store rainwater for garden water supply and leads to common breeding sites for *Aedes* mosquitoes (Ritchie et al. 2002, Duval et al. 2022). We first investigated whether the presence/absence of *Ae. albopictus* larvae in water habitats could be related to specific biotic and abiotic features. With the exception of N_2_O and CH_4_ concentrations, our results showed no significant differences in water physicochemical properties and microbial composition between colonized and noncolonized water samples. We showed that the average water temperature and pH values of colonized larval habitats were 21.6°C and 7.4°C, respectively. The temperature ranged between 15°C and 26°C, while the pH values were between 5.7 and 8.8. Consistent with the findings of other studies, the average electrical conductivity and dissolved oxygen were 0.19 S/m and 6.45 mg/l, respectively (Rao et al. 2011, Garcia-Sánchez et al. 2017, Dalpadado et al. 2022). These values ​​are optimal for the development of *Ae. albopictus* (Delatte et al. 2009, Medeiros-Sousa et al. 2018, Reinhold et al. 2018). When measured as a percentage of the calculated (or expected) amount at saturation, referred to as dissolved saturation (%DO), dissolved oxygen was on average 2.1%. This contrasted with previous measures of %DO of ~1%–1.5% in *Aedes* breeding sites (Gopalakrishnan et al. 2013, Medeiros-Sousa et al. 2020). Interestingly, it was previously shown that %DO is positively correlated with mosquito larval density and can influence the forms and toxicity of dissolved metals as well as the concentrations of certain chemicals, such as sulphides and ammonia (Khalid et al. 1978, Gopalakrishnan et al. 2013). Regarding redox potential, higher values were observed in *Ae. aegypti* breeding sites (Garcia-Sánchez et al. 2017) and could be explained by variation in microbial communities or ionic composition (DeLaune and Reddy 2005, Søndergaard 2009). With the exception of oxygen, dissolved gases in larval breeding sites remain poorly studied. Our study is the first to examine the presence and concentration of several gases in *Ae. albopictus* water habitats. Interestingly, it was shown that the presence of methane in the larval habitats of *Culex pipiens* increased oviposition, while that of CO_2_ did not seem to affect mosquito colonization or fitness (Bentley and Day 1989) or *Aedes* hatching stimulation (Judson 1960). In addition, methane and dissolved CO_2_ exposure could also affect water microbial communities (Brankovits et al. 2017). Nitrate, potassium, and chloride ions were detected at higher concentrations than those found in tap water, which could be linked to the use of nitrate fertilizers and the importance of fertilizer inputs in intensive urban agriculture (Amerasinghe et al. 1995, Panno et al. 2006, Wakejo et al. 2022). Although previous studies showed that mosquitoes are able to reproduce over a wide range of ion concentrations (Sehgal and Pillai 1970, Bashar et al. 2016, Garcia-Sánchez et al. 2017), the impact of water ionic composition on mosquito physiology remains unclear. Due to confounding factors in a complex field environment, further studies are required to investigate the impact of ionic and gas composition alone or in combination with other physicochemical parameters on different mosquito traits. For instance, the bioavailability of contaminants in water may be strongly influenced by the chemical environment (in particular ionic composition) and could in turn affect mosquito larvae by either directly modulating their physiology or indirectly modulating the microbial communities of breeding sites (Adams et al. 2020, Hery et al. 2021). Recent studies have shown that ion content in water is linked to conductance capabilities and can influence the growth rates of larvae and pupae, potentially impacting the microbial community composition in both the larval diet and water environment over time (Ukubuiwe et al. 2020, Mamai et al. 2021). Conducting controlled experiments in the laboratory could be beneficial for assessing the causality between these factors, determining whether gases and ionic compositions influence the presence of *Ae. albopictus* larvae in water habitats or whether colonization leads to differential abundance of these compounds.

Consistent with previous reports, the dominant bacterial phyla in colonized water samples were Proteobacteria (currently named Pseudomonadota), Actinobacteria (currently named Actinobacteriota), and Bacteroides (currently named Bacteroidota) (Wang et al. 2018, Scolari et al. 2021), while Ascomycota, Basidiomycota, and Cryptomycota (previously named Rozellomycota) were the dominant fungal phyla (Tawidian et al. 2021). The microalga *Picochlorum* and the protozoan flagellate *Spumela* were found to be specific to colonized waters. Interestingly, polysaccharides extracted from *Picochlorum* were shown to exhibit antiviral activity against human enterovirus 71 (Guo et al. 2021). It could be interesting to evaluate whether this effect persists in larvae and adult mosquitoes and could interfere with arbovirus transmission. Surprisingly, Egizi et al. (2015) observed the opposite tendency, with a negative correlation between the density of *Culex quinquefasciatus* larvae and that of *Spumela*. Overall, our results confirmed that a fraction of microorganisms present in larval-breeding water are maintained in the larval stage with higher microbial diversity in water-breeding sites compared to in larvae (Dada et al. 2014, Coon et al. 2016, Strand 2018, Wang et al. 2018, Scolari et al. 2021). We identified bacteria such as *Rhizobium* and fungi such as *Dacrymyces* and *Pyrenochaetopsis* that were shared between water and larvae. However, whether these taxa play a role in mosquito biology is still unknown. *Rhizobium* was listed as part of the core microbiota in different *Anopheles* mosquito tissues (Tchioffi et al. 2016, Guégan et al. 2018). Another study showed that *Ae. albopictus* females were not attracted to or repelled by *Rhizobium huautlense* isolated from canebrake bamboo, but we cannot exclude a role in larval development (Ponnusamy et al. 2015). Interestingly, noncolonized water exhibited higher fungal diversity and specific fungal composition as well as higher concentrations of N_2_O compared to colonized samples. This could be explained by the fact that N2O production is a widespread trait in fungi (Maeda et al. 2015).

Physicochemical parameters and microbial communities interact with each other in aquatic ecosystems (Bojarczuk et al. 2018, Rahman et al. 2021) and can also be influenced by urban pollution. Physicochemical factors and pollution exposure could directly alter bacterial communities by impairing microbial physiological activities or indirectly by establishing conditions that adversely affect microorganisms. In community gardens, rainwater tanks are mainly filled with rainwater and in some cases may be supplemented with tap water, which can be occasionally contaminated by organic molecules in trace quantities (Kim and Zoh 2016). Although rainwater is considered a contamination-free source, human activities, particularly in the industrial and agricultural sectors, pollute this pure form of water (Guidotti et al. 2000, Samuel et al. 2012). Over the last decade, a wide range of contaminants have been detected in surface water, including pharmaceutical, industrial, and pesticide compounds (Rosen 2007, Verlicchi et al. 2010, Yang et al. 2022). Some of them, such as microplastic particles and antibiotic residues, were shown to alter water microbial communities (Qing et al. 2020, Yang et al. 2022, Edwards et al. 2023). To the best of our knowledge, no study has characterized the presence of organic molecules in breeding sites without *a priori* information. In our study, we identified organic molecules, although uncertainties remain regarding some candidate compounds. Further studies could be conducted using a suspect screening approach to better identify organic molecules (Pinasseau et al. 2023). Among the identified organic molecules, we found pharmaceutical residues such as anti-inflammatories, hormones or antioxidants (3-tert-butyl-4-hydroxyanisole), as well as fertilizers (dimethylenetriurea) and plastics (phthalate derived). Due to their high prevalence in aquatic ecosystems, further experiments are needed to test the influence of these compounds on the physiology of *Ae. albopictus* (Yang et al. 2022). Interestingly, a previous study showed an effect of fertilizer (i.e. ammonium sulphate fertilizer) on *Aedes* and an impact on larval development, survival rates, and larval density (Mutero et al. 2004, Muturi et al. 2016, Darriet 2019). Moreover, Prud’homme et al. (2017) also revealed an impact of ibuprofen and benzo[a]pyrene on life-history traits and insecticide tolerance in *Ae. aegypti* (Prud’homme et al. 2017). More recently, Edwards et al. (2023) showed that microplastics decreased the bacterial and fungal diversity of the *Ae. albopictus* and *Ae. aegypti* microbiota (Edwards et al. 2023).

We classified the community gardens according to their pollution context, which allowed us to define pollution gradients that combined either the water physicochemical parameters and the pollution variables (e.g. proximity to pollution sources) or water organic molecule occurrence. Our results showed differential effects of pollution gradients according to the water colonization status. While no relationship between physicochemical and pollution variables and bacterial composition was evidenced in noncolonized water samples, the opposite was observed in colonized waters. Industrial pollution and ionic composition were positively correlated with OTUs belonging to the bacterial genus *Actinomycetospora* and fungal phylum *Rozellomycota*. Similarly, we found correlations between the presence of organic molecules in water-colonized samples and OTUs belonging to the bacterial family *Rhizobiaceae* and the phytopathogenic fungus *Ascochyta phaca. Actinomycetospora* is common in *Aedes* breeding sites (Zouache et al. 2022) and was found in this study to be highly prevalent in larvae. The Rhizobiaceae family contains phenotypically diverse organisms, including N_2_-fixing legume symbionts (known as rhizobia), plant pathogens, bacterial predators, and other soil bacteria (Kuzmanović et al. 2022). Interestingly, members of this family were already shown to be involved in the degradation of bisphenol A and 4-nonylphenol, both ubiquitous pollutants with oestrogenic activity in the aquatic environment (Cai et al. 2016, Gayathri and Da 2022). In noncolonized water samples, *Haematococcus* was positively correlated with physicochemical and pollution variables. Interestingly, *Haematococcus* are known to help to reduce water pollution, which may explain their presence in polluted environments (Krishnamurthi et al. 2023). We also found specific patterns highlighting correlations between the organic molecule gradient and OTUs identified as the green alga *N. aquatica* or as belonging to the fungal phylum Rozellomycota. Interestingly, Rozellomycota was also correlated with pollution related to industrial sources and specific physicochemical profiles in colonized waters. The Rozellomycota form a lineage basal or sister to fungi, the ancestor of Microsporidia (Corsaro et al. 2014). Their biodiversity is very rich but remains poorly characterized. However, we know that they are common in aquatic habitats and on pollen (Wurzbacher et al. 2014). They may play a key role in the decomposition of organic matter (Grossart et al. 2016). The few known species are all parasites, including water moulds and algae (*Rozella*), crustaceans (*Mitosporidium*), and endonuclear parasites of amoebae (*Nucleophaga* and *Paramicrosporidium*) (Chung et al. 2020, Corsaro et al. 2020, Gonçalves et al. 2022). Interestingly, *N. aquatica* was shown to have a negative effect on the larval development of *Cx. quinquefasciatus* mosquitoes (Gil et al. 2021). A study also showed their ability to reduce or remove chemical oxygen demand (used as a measurement of pollutants in water), nitrite, and phosphate from water (Ummalyma et al. 2023). Therefore, we could assume that this species could modulate water physicochemical parameters that could in turn have consequences for colonization by *Ae. albopictus*. Further studies under experimental conditions would help disentangle potential interactions between the microbial taxa found to be correlated and the level of anthropization as well as their impact on mosquito biology. Despite the influence of water properties on mosquito colonization, it is important to consider bidirectional relationships, as mosquito colonization itself can directly impact water chemistry and microbiology through various mechanisms. During their development, larvae metabolize organic compounds such as detritus and excrete nitrogenous waste products like ammonia (Clements 1992), which can affect nutrient levels and pH in the water. Ammonium appears in water as an excretory product of nitrogenous wastes (Cochran 1985, Clements 1992) or as a metabolite from microbially mediated decomposition of nitrogen-containing organic matter (Ward 2007). Microbial communities in water are involved in nutrient cycling and decomposition processes (Juma et al. 2021). The presence of larvae can influence the abundance and activity of these microbial communities, leading to changes in water chemistry (Kaufman et al. 1999). Additionally, larvae may consume certain microbes, influencing their abundance and composition in the water. Egg–laying by females has also been shown to shape bacterial communities in breeding sites (Mosquera et al. 2023). Furthermore, larvae actively move and filter water as they feed and navigate through aquatic habitats (Clements 1992). This physical disturbance can stir up sediment particles, release nutrients trapped in sediments, and enhance oxygen exchange at the water surface. These actions can modify nutrient availability, sedimentation rates, and oxygen levels, thereby impacting water chemistry. In summary, mosquito colonization can significantly affect water properties by altering nutrient levels, pH, microbial communities, and physical characteristics of aquatic habitats. Understanding these interactions is crucial for assessing the ecological impacts of mosquito populations on aquatic ecosystems.

## Conclusion

Our study confirmed that larval habitat is an important determinant of microbial community structure. It also revealed that N_2_O and CH_4_ were significantly higher in noncolonized water samples than in colonized water samples. Then, we found that pollution gradients differentially contributed to the microbial ecology of *Ae. albopictus*-colonized and *Ae. albopictus*-noncolonized water samples. The relationships between microbial communities and water quality presented here are a first and simplified step towards longitudinal sampling in the field and delimitation of the type of larval breeding sites in the urban mosaic to determine their environmental exposome. Further studies are needed to determine the influence of biotic and abiotic factors on the larval productivity of breeding sites in highly anthropized environments and their carry-over impact on adult life-history traits and vector competence. Taking advantage of the wide range of parameters identified in this study, artificial breeding sites could be replicated in laboratory settings to simulate specific field conditions. These controlled experiments will provide an opportunity to investigate whether pollution directly impacts microbial composition and subsequently influences mosquito colonization through measures of oviposition behavior and other life history traits.

## Supplementary Material

fiae129_Supplemental_Files

## Data Availability

The COI gene sequences were deposited in GenBank under the accession numbers OR391757–OR391848. All FastQ files were deposited in the EMBL European Nucleotide Archive (https://www.ebi.ac.uk/ena) under the project accession number PRJEB65881, PRJEB65912, and PRJEB65914 for, 16S, 18S, and ITS sequencing, respectively. Scripts used for analysis and figure generation are available in the following GitHub Repository (https://github.com/penelopeduval/Pollution-gradients-shape-microbial-communities-associated-with-Ae.-albopictus-larval-habitats). All other relevant data supporting the findings of this study are available within the article and its supplementary information.
