# Supplementary figures and images for: Pollution gradients shape microbial communities associated with Ae. albopictus larval habitats in urban community gardens
Source: FEMS Microbiol Ecol. 2024 Sep 26;100(11):fiae129. doi: 10.1093/femsec/fiae129 (PMC11523617; doi:10.1093/femsec/fiae129)

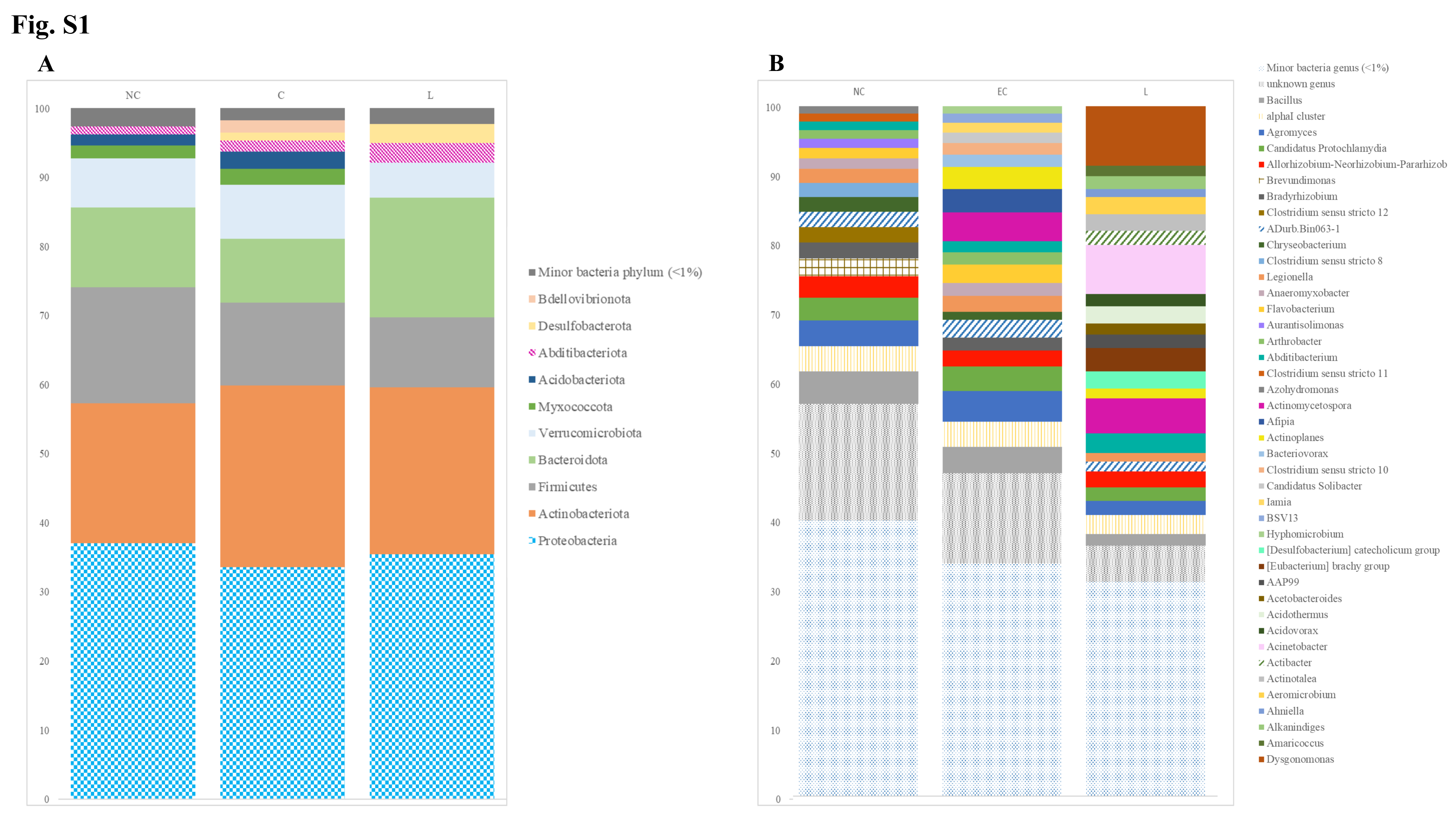

Supplement: fiae129_Supplemental_Files [file fiae129_supplemental_files.zip › Supplementary Figure 1.tif]

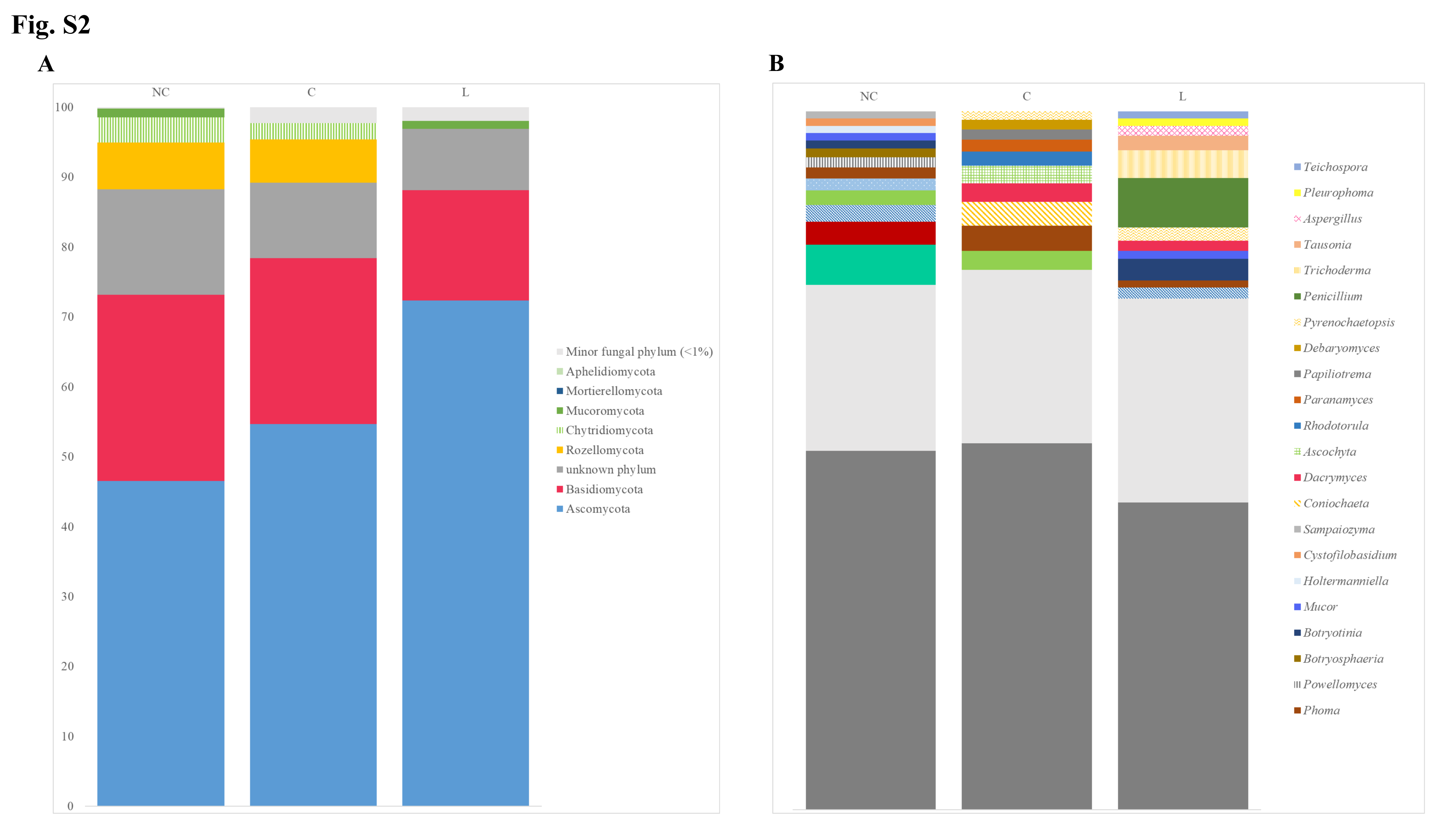

Supplement: fiae129_Supplemental_Files [file fiae129_supplemental_files.zip › Supplementary Figure 2.tif]

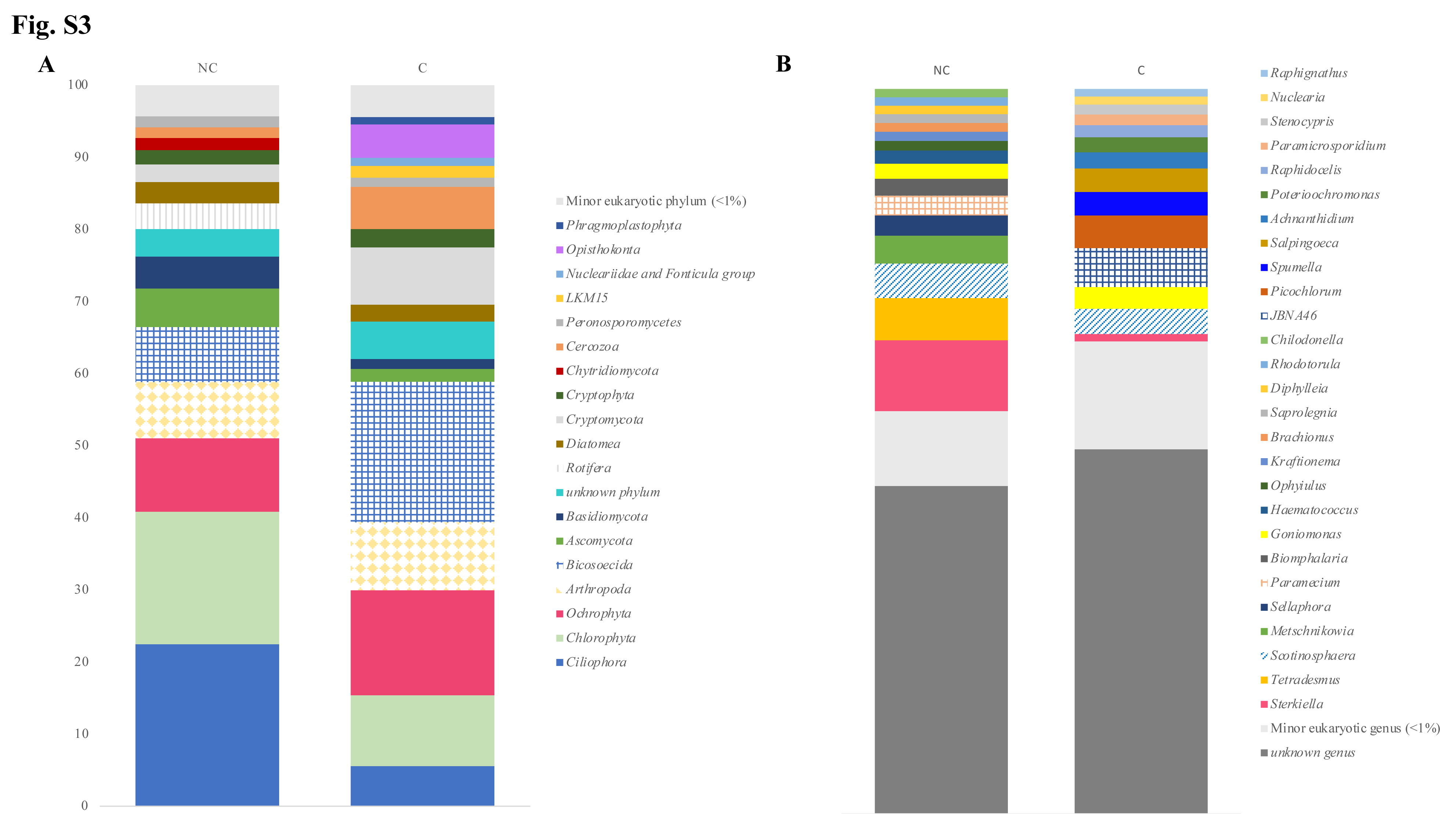

Supplement: fiae129_Supplemental_Files [file fiae129_supplemental_files.zip › Supplementary Figure 3.tif]
